# In Situ Formation of Multi-Principal Element Oxide on a Bulk Nanoporous Intermetallic Alloy for Ultra-Efficient Hydrogen Production at Ampere-Level Current Density

**DOI:** 10.1021/acsami.5c03821

**Published:** 2025-05-21

**Authors:** Xiang Gao, Wenyu Lu, Shuo Shuang, Quanfeng He, Zhaoyi Ding, Yujing Liu, Baisong Guo, Zhe Jia, Shijun Zhao, Yong Yang

**Affiliations:** † Department of Mechanical Engineering, College of Engineering, 53025 City University of Hong Kong, Tat Chee Avenue, Kowloon Tong, Kowloon, Hong Kong 999077, China; ‡ School of Materials Science and Engineering, Jiangsu Key Laboratory for Advanced Metallic Materials, 12579 Southeast University, Nanjing 211189, China; § Institute of Materials Modification and Modeling, School of Materials Science and Engineering, 12474 Shanghai Jiao Tong University, Shanghai 200240, China; ∥ Institute of Advanced Wear & Corrosion Resistant and Functional Materials, 47885 Jinan University, Guangzhou, Guangdong 523808, China; ⊥ Department of Materials Science and Engineering, College of Engineering, City University of Hong Kong, Tat Chee Avenue, Kowloon Tong, Kowloon, Hong Kong 999077, China; # Yuhua Institute of Advanced Materials, Baoji Xigong Titanium Alloy Products Co., Ltd, Baoji 721300, China

**Keywords:** eutectic multiprincipal element alloy, electro-dealloying, oxide, hydrogen evolution reaction, stability, amorphization

## Abstract

Developing highly efficient and durable electrocatalysts for hydrogen production via water splitting remains a pivotal challenge for sustainable energy. In this work, we present a bulk nanoporous C15 intermetallic alloy synthesized through electrodealloying of a eutectic multiprincipal element precursor. Unlike conventional metallic nanostructures, this catalyst features an ultrathin multiprincipal element oxide (MPEO) layer, which generates abundant active sites and achieves exceptional hydrogen evolution reaction (HER) activity, surpassing most reported catalysts. Crucially, the material demonstrates unprecedented stability at industrial-level current densities (1 A/cm^2^ at 396 mV), enabled by operando amorphization of the MPEO layer during prolonged operation. This structural evolution stabilizes the catalyst–electrolyte interface while retaining intrinsic activity. Our findings redefine design principles for robust, high-performance electrocatalysts by integrating bulk intermetallic architectures with self-optimizing surface chemistry.

## Graphical abstract

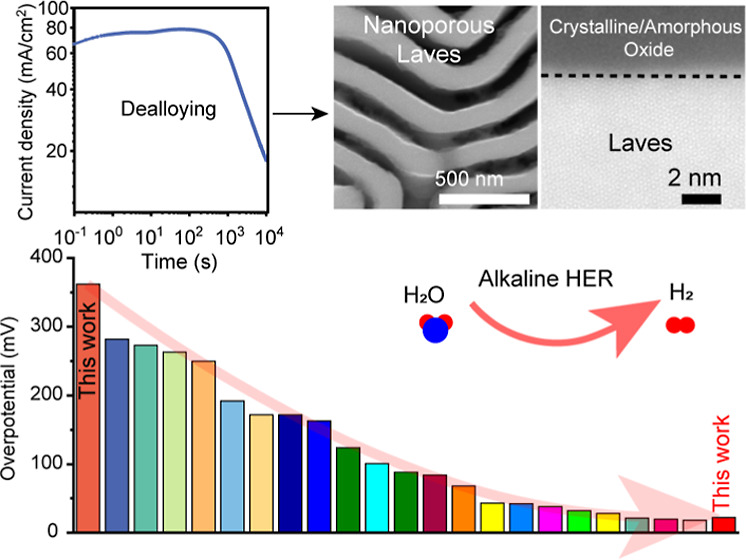

## 1 Introduction

Hydrogen is a clean and renewable energy source poised to play a pivotal role in the future energy landscape.^1^ Among the various methods for hydrogen production, alkaline Hydrogen Evolution Reaction (HER) stands out as a cost-effective technology for generating green hydrogen.^2^ Despite its potential, the market share of the alkaline HER currently remains below 5%, primarily due to the lack of highly efficient and durable electrocatalysts suitable for industrial applications.^3^ Currently, the iron triad (Fe, Co, and Ni), Cu, and Mo are the most widely used non-noble cathode catalyst for the HER owing to their natural abundance and low cost.^4–6^ Nevertheless, these materials exhibit relatively modest catalytic activity compared to Platinum Group Metal (PGM) electrocatalysts,^7^ which has significantly hindered the development of the alkaline HER.

Recently, electrochemical dealloying has emerged as a promising method to enhance catalytic performance by creating nanostructured electrocatalysts, which can increase the number of active sites.^8–11^ While significant progress has been made in improving the catalytic activity of electrocatalysts through dealloying, the fundamental mechanisms behind this enhancement remain unclear. Typically, dealloying introduces oxides, yet most analyses of electrocatalysis focus on the metallic state for simplicity.^12–19^ Additionally, the surface reconstruction mechanisms during cyclic voltammetry (CV) activation and long-term HER operation are rarely explored, despite their crucial role in determining electrocatalytic activity and stability.^20^

In this study, we successfully fabricated a bulk nanoporous C15 Laves-phase intermetallic material by dealloying an eutectic multiprincipal element alloy (EMPEA) precursor. Notably, this nanostructured intermetallic underwent surface oxidation following dealloying and displayed outstanding HER performance, surpassing a wide range of both precious and nonprecious metallic electrocatalysts reported to date.^10^ Moreover, our nanoporous intermetallic electrocatalyst showcased remarkable stability, sustaining high electric current densities of up to 1 A/cm^2^ at ampere-level conditions, a critical attribute for industrial-scale applications.^21,22^ These findings pave a path for the practical deployment of bulk nanoporous intermetallics in industrial settings, offering sustainable and efficient solutions to key challenges in energy conversion processes.

## 2 Results and Discussion

### 2.1 Structural Characterization of the Alloy Precursor and Dealloyed Nanostructures

Using the arc-melting technique, we synthesized an EMPEA precursor with a composition of FeCoNiNb_0.5_ (in atomic percent) (see Section 4). Transmission electron microscopy (TEM) analysis depicted in Figure 1a revealed that the as-cast sample (AC) comprised a face-centered cubic (FCC) phase alongside a C15 Laves phase, a finding corroborated by X-ray diffraction (XRD) patterns (Figure S1a). Initially, we explored the electrochemical response of the EMPEA in 1 M HCl, observing active dissolution beyond the corrosion potential (∼−0.28 V vs saturated calomel reference electrode (SCE)) without passivation (Figure 1b). Subsequently, dealloying was conducted at 0.1 V vs SCE for 10^4^ s (Figure 1c), within the realm of active dissolution of the FCC phase while avoiding severe corrosion of the Laves phase and oxidation of water or chloride. This process led to the rapid formation of a porous C15 nanostructure, as evidenced by the XRD patterns (Figure S1a) and scanning electron microscopy (SEM) imagining, indicating selective dissolution of the FCC phase (inset of Figure 1c).

**Figure 1:**
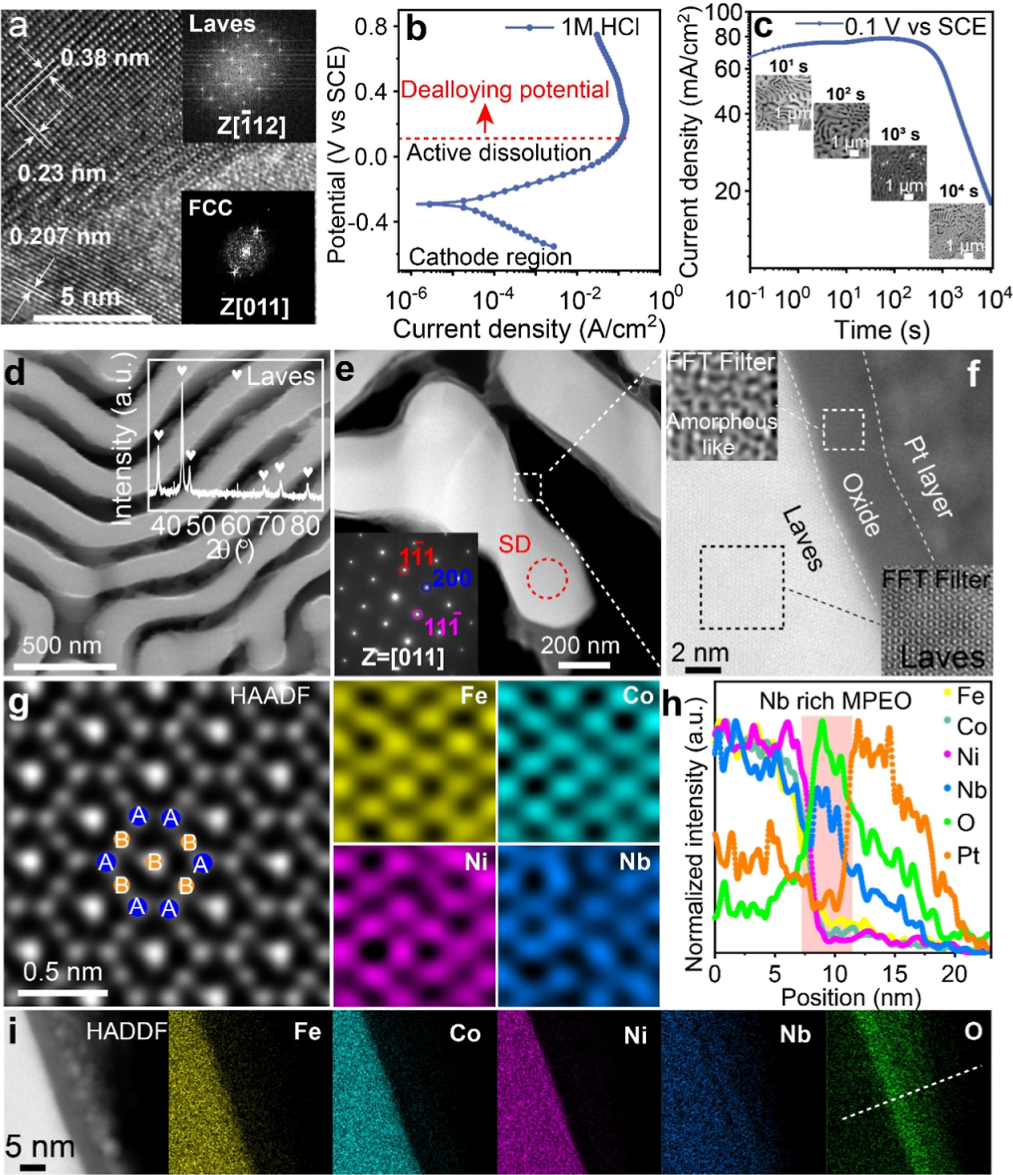
Characterization of the as-cast EMPEA and 10^4^ s dealloyed EMPEA. (a) High-resolution transmission electron microscopy (TEM) image of the dual phase eutectic structure; the insets show the Fast Fourier Transform (FFT) images of Laves and FCC. (b) Potential dynamic polarization (PDP) curve of the as-cast eutectic alloy in 1 M HCl. (c) Potentiostatic polarization curve at 0.1 V vs SCE in 1 M HCl; insets show Secondary Electron Microscopy (SEM) images of the EMPEA at different dealloying times. (d) SEM image of the EMPEA after 10^4^ s dealloying; the inset shows the GXRD. (e) Low-magnification TEM image of the dealloyed EMPEA; the inset shows the SADP. (f) High-magnification TEM image of the dealloyed EMPEA; insets show the corresponding FFT filter images of Laves and oxide. (g) High-magnification HAADF-STEM image accompanied by atomic-resolution elemental maps clearly showing the ordered crystallographic structure and site occupancy of the Laves structure. (h) EDS line scan profile in (i) confirms that the amorphous-like layer is multicomponent, but Nb- and O-rich. (i) Elements maps of Fe, Co, Ni, Nb, and O.

To delve deeper into the selective dissolution dynamics of the EMPEA, we prepared single-phase FCC (Fe_35.3_Co_29.41_Ni_29.41_Nb_5.88_) and Laves (Fe_20.33_Co_31.25_Ni_24.87_Nb_23.55_) samples, mirroring the two phases present in the EMPEA, as validated by their respective XRD patterns (Figure S1b). Comprehensive electrochemical characterizations encompassing open-circuit potential (OCP), Nyquist plots, potentiodynamic polarization curves, and potentiostatic polarization curves in a standard three-electrode cell collectively indicated a substantial thermodynamic driving force and a pronounced kinetic contrast in the dealloying process between the FCC and Laves phases (Figure S1c–f). Furthermore, the kinetics of dealloying were scrutinized by tracking dealloying depths (inset of Figure S2a) at varying time intervals under a constant dealloying potential of 0.1 V vs SCE. Figure S2a illustrates a scatter plot of dealloying depth against dealloying time on a double logarithmic scale for the as-cast (AC) EMPEA. Through fitting our experimental data to the well-established theoretical models,^23^ we deduced that the selective dissolution mechanism in our EMPEA involves a multiscale process encompassing both interface-driven and long-range diffusion processes.^24^

By following the electrochemical dealloying method described above, we successfully fabricated a bulk nanoporous structure with an average ligament width of 165 nm (Figures 1d and S3). To characterize the atomic structure, we first conducted grazing X-ray diffraction (GXRD), where we pinpointed peaks corresponding to a C15 Laves phase (inset of Figure 1d). Subsequently, utilizing the focused ion beam (FIB) technique (see Section 4), we prepared TEM samples and observed the presence of the C15 crystalline structure inside the ligament (inset of Figure 1e). Apart from the C15 Laves phase, we also detected a layer of multiprincipal element oxide (MPEO) on the surfaces of the ligament. The fast Fourier transform­(FFT) filtered image of the MPEO layer unveiled an amorphous-like structure atop the C15 Laves phase (inset of Figure 1f), with a thickness of less than 5 nm. A similar occurrence of surface oxidation during corrosion studies was reported in ref 25. For a detailed chemical analysis of the C15 phase, we conducted high-resolution, high-angle annular dark-field (HAADF) imaging and elemental mapping, revealing the atomic-scale distribution of Fe, Co, Ni, and Nb (Figure 1g). Notably, Fe, Co, and Ni share a similar sublattice, while Nb occupies a distinct sublattice, forming an AB_2_-type Laves phase. Furthermore, line scans (Figure 1h) and elemental mapping (Figure 1i) demonstrate that the MPEO surface layer is enriched in Nb and O, with relatively lower concentrations of Fe, Co, and Ni. Additionally, we analyzed the surface chemistry of the bulk nanoporous structure using X-ray photoelectron spectroscopy (XPS) (see Section 4, Figures S4 and S5). Consistent with the TEM observations, the XPS spectra of Fe, Co, Ni, and Nb demonstrated clear signs of surface oxidation, in contrast with more metallic states detected internally. Through data fitting (Figure S4), we measured the relative concentrations of Fe, Co, Ni, Nb, and O over different etching times (Figure S5). The XPS assessment confirmed that the surface composition of our bulk nanoporous structure is enriched in Nb and O, while showing a deficiency of Fe, Co, and Ni, aligning with our TEM results (Figure 1h,i). Moreover, X-ray absorption spectroscopy (XAS) was performed on the nanoporous structure as well as a Nb foil as a reference material (Figure S6). Owing to the enrichment of Nb, the Nb K edge threshold energy and maximum energy for the X-ray absorption by our nanoporous structure look quite similar to those of Nb foil (Figure S6a,b). Aside from Nb–Nb bonds, a few Nb–O/M bonds were observed on the surface of the nanoporous structure (Figure S6c,d and Table S1).

### 2.2 Electrocatalytic Performance of the Nanoporous MPEA

To assess the electrocatalytic properties of our nanoporous MPEA, we set up a three-electrode system in 1 M KOH (see Section 4). Before evaluating the HER performance, we activated our porous MPEA using cyclic voltammetry (CV) within the range of −0.9 V to −1.3 V vs Hg/HgO for 200 cycles. Surprisingly, we observed a dramatic enhancement in the HER activity following CV activation (Figure S7a). After the CV activation, we evaluated the HER electrocatalytic performance of our nanostructured MPEA in 1 M KOH, using commercial nickel foam (NF) as a benchmark. Initially, we measured the linear sweep voltammetry (LSV) curves of our nanostructured MPEA obtained at various dealloying time durations, as illustrated in Figure 2a. Our results reveal that with increasing dealloying time, the nanoporous MPEA exhibits significantly superior catalytic activity compared to commercial NF. Notably, the activated nanoporous MPEA achieved an exceptionally low overpotential (η_10_) of just 22 mV at 10 mA/cm^2^, significantly outperforming commercial NF (η_10_ = 273 mV) (Figure 2b) and even Pt/C.^10^ The Tafel slope of our nanoporous MPEA decreased from 116 to 77 mV/dec with increasing dealloying time (Figure 2c), indicating a transition in the rate-determining step­(RDS) from the Volmer reaction to a mixed Volmer–Heyrovsky pathway. Electrochemical impedance spectroscopy (EIS) further corroborated the superior HER performance of our nanoporous MPEA compared to that of commercial NF (Figure S9, Table S2), with a much smaller charge transfer resistance.^26,27^

**Figure 2:**
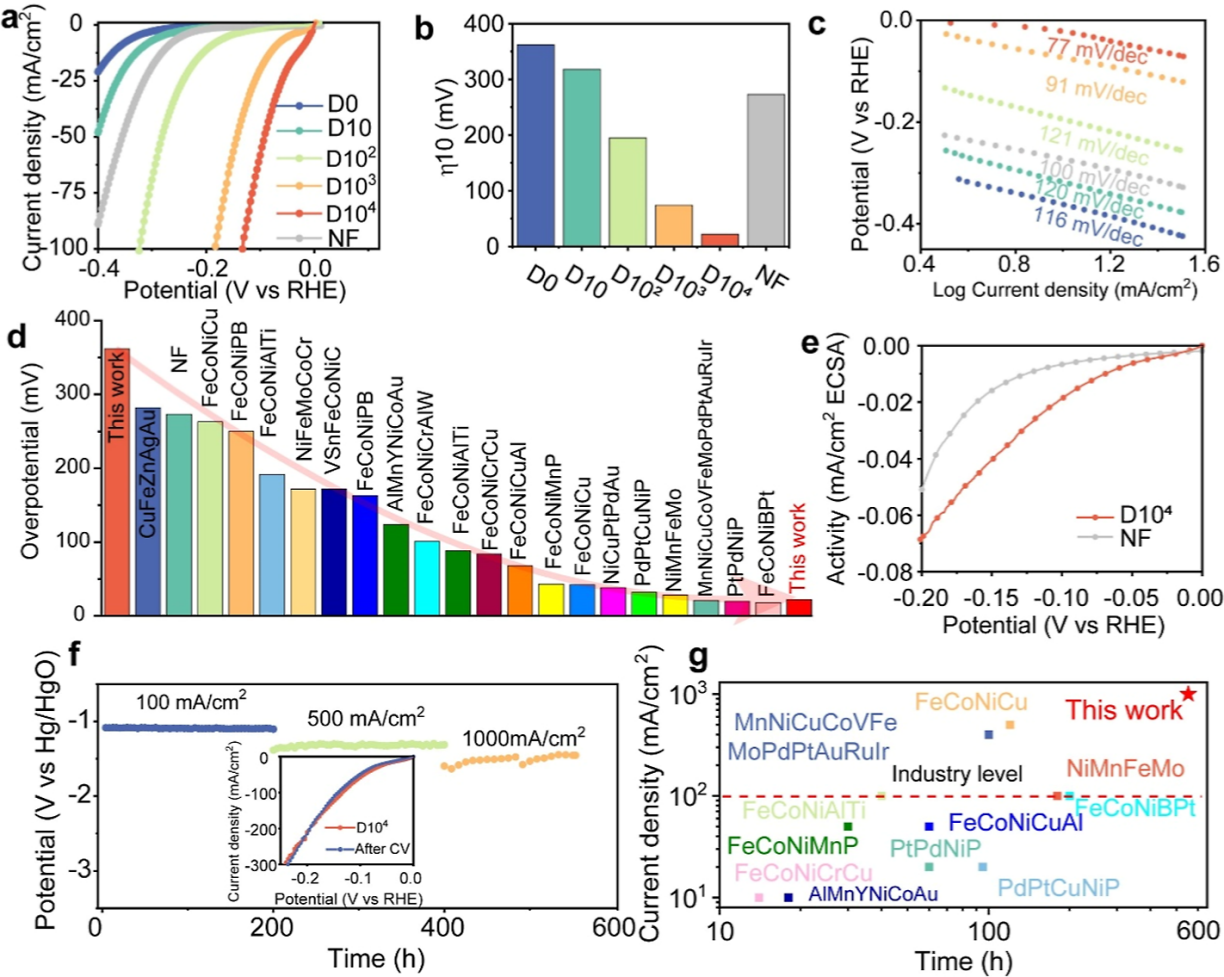
Alkaline HER performance of the nanoporous MPEA at different dealloying times. (a) Linear sweep voltammetry­(LSV) curves. (b) Overpotential at 10 mA/cm^2^. (c) Tafel slopes. (d) Comparison of our nanostructured MPEA and other HER electrocatalysts in terms of overpotential at 10 mA/cm^2^. (e) Specific activity comparison of the 10^4^ s dealloyed nanostructure and NF. (f) Stability test of the 10^4^ s dealloyed nanostructure at the current density of 100 mA/cm^2^, 500 mA/cm^2^, and 1000 mA/cm^2^ respectively; the inset is the LSV curves before and after CV stability. (g) Stability comparison of our nanoporous MPEA and other HER electrocatalysts in terms of tested time and current density.

We conducted a comparative analysis of our nanoporous MPEA against known electrocatalysts, as detailed in Table S3, focusing on key metrics such as η_10_. Notably, as illustrated in Figure 2d, our nanoporous MPEA stands out, surpassing the performance of most platinum group metal (PGM) catalysts. To unveil the inherent activity of our nanoporous MPEA, we normalized the current density by the electrochemical surface area (ECSA),^28^ as depicted in Figure S10. The specific activity of our nanoporous MPEA, showcased in Figure 2e, notably exceeds that of commercial NF. Moreover, we assessed the HER performance of the nanoporous MPEA following further dealloying (e.g., with a dealloying duration of 20,000 s) and noted a comparable activity level to that of the nanoporous structure after the 10^4^ s dealloying (Figure S11). This departure from conventional dealloying studies, which often report performance deterioration,^13,14,29^ may be attributed to the excellent corrosion resistance of our nanoporous MPEA, characterized by the protective amorphous-like Nb rich oxide layer shielding the nanosized ligaments (Figure 1). Additionally, efficient detachment of hydrogen bubbles from the electrocatalyst surface is crucial for the HER. Our observations at a current density of 500 mA/cm^2^ (Video S1) reveal that bubbles rapidly detach from the catalyst surface with minimal accumulation, ensuring uninterrupted active site availability and sustained catalytic activity. Furthermore, contact angle measurements (Figure S9a) demonstrate a significant shift in surface wettability after dealloying—transitioning from poorly wetting (hydrophobic) to hydrophilic behavior. This enhanced wettability may stem from the formation of surface complex oxide films during dealloying, which not only improves electrolyte penetration but also maximizes active site exposure. Such structural characteristics facilitate efficient bubble release and promote rapid mass transport, both of which are critical for optimizing the HER performance.

In addition to catalytic activity, we assessed the electrochemical stability of our nanoporous MPEA at current densities of 100 mA/cm^2^, 500 mA/cm^2^, and 1000 mA/cm^2^ over approximately 600 h (Figure 2f). Remarkably, no potential amplification was observed during the long-term, high-current experiments, hence indicative of excellent stability. Comparative analysis of the stability of our nanoporous MPEA under varying operating current densities and timeframes (Table S3), as illustrated in Figure 2g, demonstrates that our nanoporous MPEA is among the most durable electrocatalysts reported to date. Additionally, we performed the CV stability tests over 10,000 cycles, and the LSV curves obtained before and after the CV stability tests almost overlap, which further corroborates the exceptional stability of our nanoporous MPEA (inset of Figure 2f). Accordingly, ICP-OES of the KOH solution after the CV stability tests showed a low Nb content and negligible Fe, Co, and Ni content (Figure S12e).

### 2.3 Surface Reconstruction on the Nanoporous MPEA

To investigate the underlying mechanisms, we first systematically analyzed our nanoporous MPEA after CV activation. The postactivation surface morphology remained largely consistent with the preactivation state (Figure 3a). Grazing-incidence X-ray diffraction (GXRD) analysis revealed peaks corresponding to a C15 Laves phase (inset of Figure 3a), confirming the surface crystal structure. TEM observations further supported the presence of the C15 Laves phase, as evidenced by the selected area diffraction pattern­(SADP) (inset of Figure 3b). However, the surface MPEO exhibited a crystalline structure, as indicated by the FFT image (inset of Figure 3c). Comprehensive surface chemistry analysis, including line scans (Figure 3d), elemental mapping (Figure 3g), and TEM–EDS (Figure S7g), consistently demonstrated that the post-CV activation surface MPEO contained Fe, Co, Ni, Nb, and O. XPS analysis further confirmed the formation of a multicomponent surface oxide with a relatively high O content (Figures 3e, S7, and S8). In contrast, inductively coupled plasma optical emission spectrometry (ICP-OES) (Section 4) of the KOH solution after CV activation revealed a high Nb content, suggesting the dissolution of Nb-rich MPEO into the KOH solution during CV activation (Figure 3f). This notable shift in surface chemistry is likely due to Nb’s resistance to acidic conditions but solubility in alkaline environments.^30^ Based on these findings, we conclude that CV activation led to the formation of a crystalline MPEO on the surface of our nanoporous MPEA.

**Figure 3:**
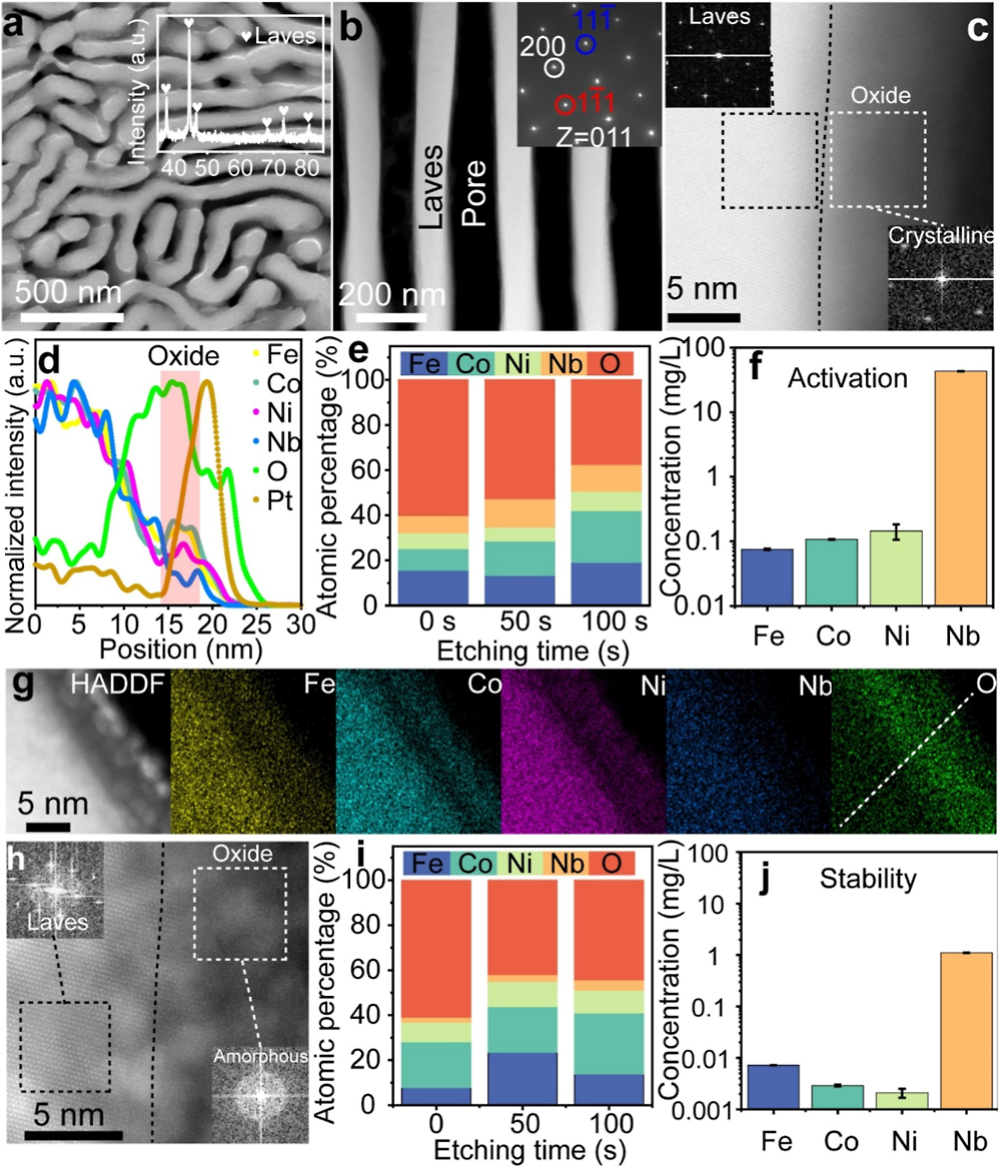
Structural characterization of the 10^4^ s dealloyed EMPEA after CV activation and stability in 1 M KOH. (a) SEM images of the eutectic alloy after dealloying and CV activation; the inset shows the GXRD. (b) Low-magnification TEM image of the dealloyed eutectic alloy after CV activation; the inset shows the SADP. (c) High-magnification TEM image; insets show the corresponding FFT images of the oxide and Laves phase. (d) EDS line scan profile in g confirms that the crystalline oxide layer is multicomponent, oxygen-rich. (e) Relative atomic concentration of Fe, Co, Ni, Nb, and O with etching time after CV activation, as obtained from the quantitative analysis of the XPS. (f) ICP-OES of the KOH electrolyte after CV activation. (g) Elements maps of Fe, Co, Ni, Nb, and O. (h) High-magnification TEM image after stability; insets show the corresponding FFT images. (i) Relative atomic concentration of Fe, Co, Ni, Nb, and O with etching time after stability, as obtained from the quantitative analysis of the XPS. (j) ICP-OES of the KOH electrolyte after stability.

To further understand the origins of the exceptional stability, we systematically evaluated the performance of our nanoporous MPEA operating at 100 mA/cm^2^ for 50 h. Remarkably, poststability testing revealed an improvement in HER performance compared to the initial state, rather than degradation (Figure S12). This enhancement is likely due to further dealloying, as indicated by the significantly increased ECSA of 176.8 mF/cm^2^, which far exceeds that of the initial nanoporous structure. Comprehensive morphological, structural, and compositional analyses were also conducted. As shown in Figure S13, the surface morphology became rougher after stability testing than its state following dealloying or CV activation, while the C15 Laves phase crystal structure within the ligament remained intact (inset of Figure S13a). TEM observations indicated that while the C15 Laves phase persisted within the ligament, the surface MPEO phase became amorphous (Figure 3h). A similar amorphization process during electroreduction reactions has been recently reported in the literature.^31,32^ XPS analyses provided further insights into the dealloying process of the MPEO during stability testing (Figures 3i, S14, and S15). Additionally, ICP-OES analysis of the KOH solution poststability testing revealed an enrichment of Nb (Figure 3j), further supporting the dealloying of Nb during the prolonged stability tests. Collectively, these findings suggest that the outstanding stability of our nanoporous MPEA can be attributed to amorphization of the MPEO surface layer, which enhances its durability and catalytic performance under operational conditions.

### 2.4 Atomistic Mechanisms for Enhanced Electrocatalytic Performance

The catalytic potential of intermetallic compounds has been widely investigated in the literature, primarily due to their ordered atomic structures and tunable catalytic sites.^3,13,15,19,21,22,33,34^ However, prior research has predominantly focused on intermetallics, often overlooking the formation of oxide layers on their surfaces during electrochemical processes. In contrast to the conventional findings, our study has uncovered the presence of both crystalline and amorphous MPEO layers surrounding the C15 intermetallics following CV activation and stability testing, as illustrated in Figure 3. A thorough understanding of the properties and behavior of these MPEO layers is crucial for the development of highly efficient and durable catalysts specifically designed for the HER.

To explore the fundamental mechanisms at play, we conducted an extensive set of density functional theory (DFT) calculations to investigate the surface adsorption properties of both crystalline and amorphous MPEOs (see Section 4). Notably, our DFT analyses unveil a striking similarity: both crystalline and amorphous MPEOs exhibit more negative absorption energies for water than Pt(111), indicating a strong and favorable absorption of H_2_O molecules onto the MPEO surfaces, irrespective of their atomic structure (Figure 4a). Additionally, we illustrate the charge density differences before and after water adsorption in Figure 4b,c, where yellow regions represent charge accumulation and blue regions denote charge depletion. Intriguingly, our calculations show negligible charge transfer between Nb atoms and H_2_O molecules, while significant charge transfer occurs between Fe, Co, and Ni atoms and H_2_O molecules. This observation hints that the strong water adsorption observed in our MPEO primarily stems from the presence of active Fe, Co, and Ni sites within both the crystalline and amorphous atomic structures.

**Figure 4:**
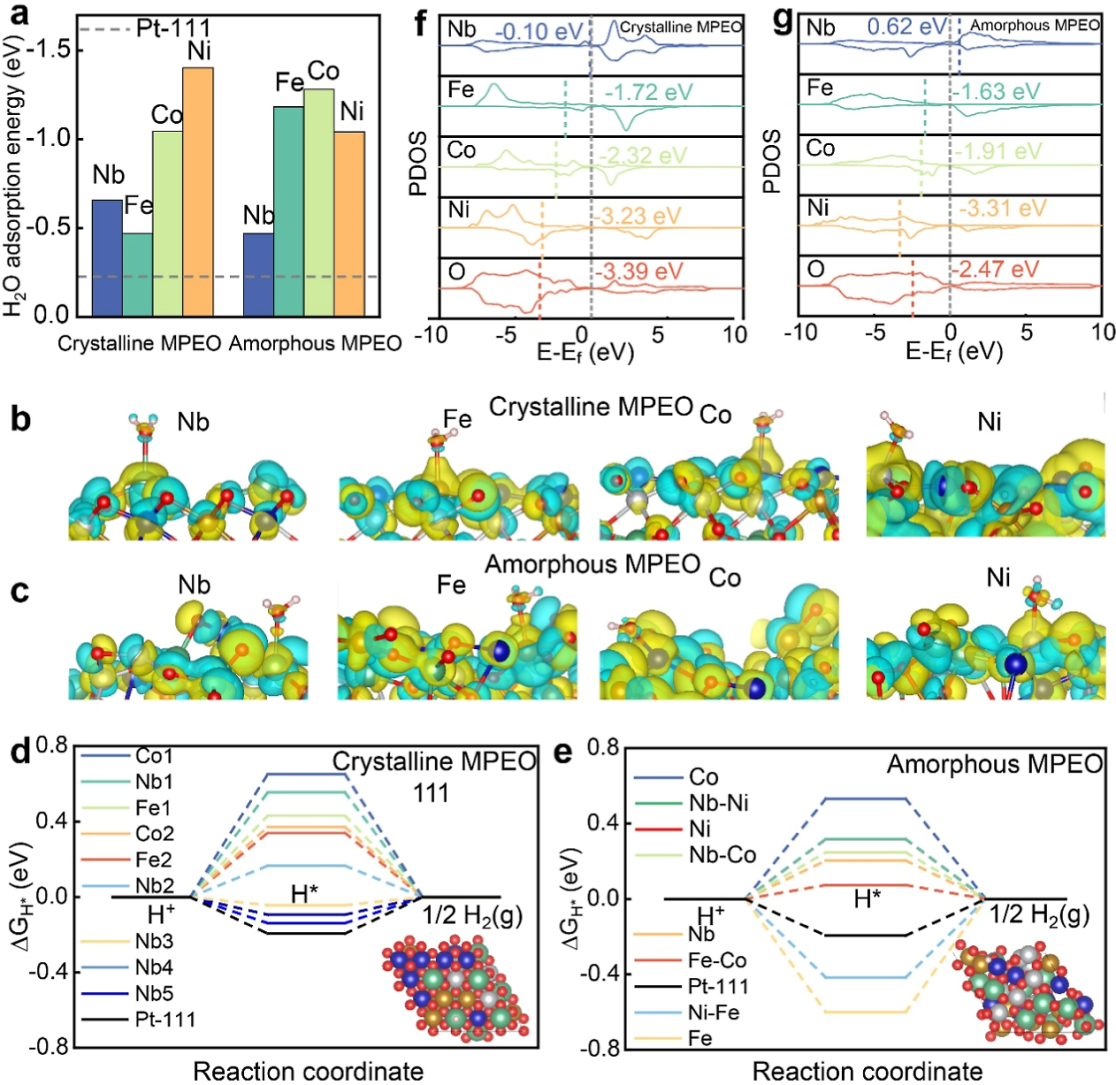
The atomic and electronic origin of the excellent HER activity in our crystalline MPEO and amorphous MPEO. (a) DFT calculated adsorption energies of H_2_O molecules on the surfaces of the crystalline and amorphous MPEOs, compared to that on the Pt(111) surface. (b,c) The charge density difference before and after the adsorption of H_2_O in crystalline and amorphous MPEOs. The blue and yellow isosurfaces correspond to charge densities of 0.01 e/Å^3^ and −0.01 e/Å^3^, respectively. (d,e) Adsorption free energy versus the reaction coordinate of the HER in crystalline and amorphous MPEOs, respectively. The insets show the local atomic environments of the optimal hydrogen adsorption active sites at the (111) surface. (f,g) Projected density of states (PDOS) for the d orbitals of each constituent metal in crystalline and amorphous MPEOs, respectively.

Subsequently, we investigated the adsorption and desorption behaviors of hydrogen atoms by calculating their adsorption energies. In the crystalline MPEO model, we focused exclusively on the top sites of various metal atoms for hydrogen adsorption as the bridge and hollow sites are typically occupied by oxygen atoms. Our results reveal that Nb sites exhibit hydrogen adsorption energies closest to zero, while Fe, Co, and Ni sites show limited favorable absorption characteristics (Figure 4d, Table S4). In contrast, the amorphous MPEO structure demonstrates additional favorable adsorption sites for Fe, Co, and Ni at both the top and bridge positions, with adsorption energies nearing zero (Figure 4e, Table S5). This improved hydrogen adsorption at Fe, Co, and Ni sites in the amorphous MPEO compensates for Nb dissolution, thereby amplifying the overall HER performance. Further analysis using projected density of states (PDOS) calculations reveals that the d-band centers of Fe and Co in the amorphous MPEO shift closer to the Fermi level than those in the crystalline MPEO (Figure 4f,g). This shift enhances the hydrogen adsorption capabilities of Fe and Co sites, resulting in adsorption energies closer to zero and highlighting the superior HER performance of the amorphous MPEO.

## 3 Conclusions

In summary, we have harnessed the process of dealloying to create a bulk nanoporous structure with an MPEO layer on a C15 MPEA. This unique architecture demonstrates exceptional water adsorption and optimal hydrogen absorption properties, rendering it highly effective for the alkaline HER. Furthermore, the operando amorphization driven by dealloying during prolonged operation enhances the electrocatalyst’s stability by generating new active sites. Our findings offer critical insights into the design and fabrication of highly efficient and durable multicomponent nanoporous electrocatalysts. These results hold significant promise for advancing the development of MPEO and intermetallic electrocatalysts in energy conversion technologies.

## 4 Experimental Section

### 4.1 Sample Preparation

The polycrystalline samples of the EMPEA FeCoNiNb_0.5_, single-phased FCC (Fe_35.3_Co_29.41_Ni_29.41_Nb_5.88_) and Laves (Fe_20.33_Co_31.25_Ni_24.87_Nb_23.55_) (atom %), were prepared through arc melting in a high-purity argon atmosphere. The purities of the raw materials for each element were at least 99.9 wt %. The ingots were remelted at least seven times to ensure the chemical homogeneity and then suction-cast into a copper plate mold. The plate mold was 6 × 10 × 60 mm^3^. Afterward, the samples were cut into the geometry of 5 × 10 × 0.8 mm^3^, followed by grounding with SiC up to 5000 grit and polishing with 0.5 μm diamond powder for further characterization.

### 4.2 Structural and Chemical Composition Characterization

The microstructure characterization was performed with SEM (Quanta FEG 450). The structural analysis of the as-cast samples was performed with conventional field emission TEM (JEM-2100, JEOL) at an acceleration voltage of 200 kV. Microstructural analysis of the dealloyed samples was conducted by a double aberration-corrected transmission electron microscopy (AC-TEM, FEI Themis Z) with energy-dispersive X-ray spectroscopy (EDS). The TEM samples were prepared by using focused ion beam (FIB) milling and lift-out techniques, with a protective layer of platinum or carbon applied to the surface. The binding energies of each element were obtained by using X-ray photoelectron spectroscopy (XPS, Thermo Scientific K-Alpha). We employed an Agilent 720ES (OES) to determine the concentration of ion Fe, Co, Ni, and Nb after cyclic voltammetry (CV) activation and stability tests.

### 4.3 Dealloying

Open-Circuit Potential (OCP), Potentiodynamic Polarization (PDP), and Electrochemical Impedance Spectroscopy (EIS) were performed in a typical three-electrode cell, which consisted of the test sample as the working electrode, a saturated calomel reference electrode (SCE), and a carbon rod counter electrode. The corrosion tests were carried out in 1 M HCl at 25 °C under atmospheric pressure using the Vertex electrochemical workstation (IviumStat Technology). The electrochemical dealloying was carried out in 1 M HCl at constant potential 0.1 V vs SCE at different times 10, 100, 1000, 10,000, and 20,000 s. The kinetics was investigated by dealloying depth measurement at different dealloying times and Cyclic Voltammetry (CV) scan between −0.1 and 0.2 V vs SCE.

### 4.4 Hydrogen Evolution Reaction

In a typical HER evaluation, the freestanding MPEA sheet (500 μm thick) is employed directly as the working electrode, either in its as-synthesized state or after dealloying, eliminating the need for secondary substrates or binders. This substrate-free approach mirrors methodologies in prior studies,^14,19,35^ where self-supported catalysts (e.g., freestanding foils, films) were tested as integrated systems to preserve structural continuity and avoid performance distortions from external interfaces. Commercial Nickel Foam with 500 μm thickness was also evaluated for comparison. We performed all measurements at room temperature in a standard three-electrode system using a H_2_-saturated 1 M KOH electrolyte. We used a carbon rod (diameter = 6 mm) as the counter electrode and a standard Hg/HgO electrode as the reference electrode. We calibrated the Hg/HgO to the reversible hydrogen electrode (RHE) under a H_2_-saturated electrolyte, with Pt foils serving as both the working electrode and counter electrode (Figure S16). We conducted electrochemical impedance spectrum measurements at an overpotential of 100 mV vs RHE with a 10 mV AC potential, ranging from 10^5^ to 0.01 Hz. We collected the time-dependent potential curve by maintaining the current density at 100, 500, and 1000 mA/cm^2^ for around 200 h, respectively. Besides, the stability was also evaluated with cyclic voltammetry for 10,000 cycles within the potential range −0.9 to −1.3 V vs Hg/HgO. To evaluate the electrochemical active surface area (ECSA), CV was carried out within the non-Faradaic potential region from −0.9 V to −0.8 V vs Hg/HgO at scan rates 10, 20, 40, 60, 80, and 100 mV/s. The ECSA was obtained according to C_dl_/C_s_, where *C*_dl_ is the measured double-layer capacitance and *C*_s_ is the specific capacitance of the flat smooth electrode surface. Here, *C*_s_ was assumed to be 0.040 mF cm^–2^ in a 1 M KOH solution.

### 4.5 Theoretical Calculations

In this study, three types of atomic models were constructed (Figure S17): C15 intermetallics, C15 crystalline MPEO, and amorphous MPEO. The C15 intermetallic and C15 crystalline MPEO structures were modeled by using the AB_2_ configuration, where Nb atoms occupied the A sites while Fe, Co, and Ni atoms were randomly distributed in equimolar ratios at the B sites. Amorphous MPEO configurations were obtained through the liquid-quenching method,^36^ where crystalline MPEO structures were initially heated to 3500 K and subsequently quenched to 300 K with a time step of 2 fs. First-principles calculations were carried out using the Vienna ab initio simulation package (VASP) based on density functional theory (DFT).^37^ The Kohn–Sham equation was solved via the projector augmented wave (PAW) approach.^38^ The exchange–correlation interactions were modeled using the Perdew–Burke–Ernzerhof (PBE) form.^39^ Hubbard-U corrections were applied to Fe, Co, and Ni, with effective *U* values of 4.00, 3.30, and 6.40 eV, respectively.^40^ Gaussian smearing with a smearing width of 0.2 eV was utilized to handle the partial orbital occupancies. A Monkhorst–Pack grid of 3 × 3 × 1 was used for sampling the Brillouin zone during structural optimization, with a kinetic energy cutoff of 500 eV for the plane waves. The electronic self-consistency loop was converged to 10^–5^ eV, while atomic forces were relaxed until the components were below 0.03 eV/Å. The surface model was simulated with a box containing 48 and 96 atoms along the ⟨111⟩ direction for C15 intermetallics and C15 crystalline MPEOs, respectively. The vacuum spacing in a direction perpendicular to the plane of the structure was 12 Å. The adsorption energies were determined by the following expression

$$E_{ads}=E_{ad/sub}-E_{ad}-E_{sub}$$

where *E*_ad/sub_, *E*_ad_, and *E*_sub_ are the total energies of the optimized adsorbate/substrate system, the adsorbate in the structure, and the clean substrate, respectively. The Gibbs free energies were calculated by

ΔG=ΔE+ΔZPE−TΔS

where the Δ*E*, ΔZPE, *T*, and Δ*S* are the free energy, total energy, zero-point energy, and entropic contributions, respectively. The vibrational entropy of hydrogen in the adsorbed state is assumed to be negligible, allowing the entropy difference Δ*S* to be approximated as

$$\Delta S=S_{H^{*}}-\frac{1}{2}S_{H_{2}}\approx \frac{1}{2}S_{H_{2}}$$

where *S*_H_2__ is the entropy of molecular hydrogen in the gas phase at standard conditions. Similarly, the zero-point energy shift can be calculated as

$$\Delta ZPE={ZPE}_{H^{*}}-\frac{1}{2}{ZPE}_{H_{2}}$$

As a result, the free energy of hydrogen adsorption is approximated by the simplified equation^41^

$${\Delta G}_{H^{*}}={\Delta E}_{H^{*}}+0.24\;eV$$

C15 intermetallics were performed as a theoretical reference (Figure S18). While this model shows favorable water adsorption and moderate hydrogen adsorption, they represent an idealized system that differs from the MPEO surfaces observed experimentally.

## Supplementary Material





## Notes

Thermodynamic and kinetic analysis of MPEA dealloying, along with structural and chemical characterization of the dealloyed nanostructure following activation and stability test, evaluation of hydrogen evolution reaction (HER) activities, ECSA estimation, DFT calculations, and atomistic models (PDF)
Video demonstration of hydrogen bubble detachment on the dealloyed nanostructure at a current density of 500 mA/cm^2^ (MP4)
