# Supplementary material for: In Situ Formation of Multi-Principal Element Oxide on a Bulk Nanoporous Intermetallic Alloy for Ultra-Efficient Hydrogen Production at Ampere-Level Current Density
Source: ACS Appl Mater Interfaces. 2025 May 21;17(22):32392–9. doi: 10.1021/acsami.5c03821 (PMC12147075; doi:10.1021/acsami.5c03821)
Supplement: Supplementary file 1 [file am5c03821_si_001.pdf]

Supporting Information

In-Situ Formation of Multi-Principal Element Oxide on Bulk Nanoporous Intermetallic Alloy for Ultra-Efficient Hydrogen Production at Ampere-Level Current Density

Xiang Gao[1, †], Wenyu Lu[1, †], Shuo Shuang[1], Quanfeng He[3], Zhaoyi Ding[1], Yujing Liu[6], Baisong Guo[4], Zhe Jia[2, *], Shijun Zhao[1, *], Yong Yang[1, 5, *]

[1] Department of Mechanical Engineering, College of Engineering, City University of Hong Kong, Tat Chee Avenue, Kowloon Tong, Kowloon, Hong Kong 999077, China

[2] School of Materials Science and Engineering, Jiangsu Key Laboratory for Advanced Metallic Materials, Southeast University, Nanjing 211189, China

[3] Institute of Materials Modification and Modeling, School of Materials Science and Engineering, Shanghai Jiao Tong University, Shanghai 200240, China

[4] Institute of Advanced Wear & Corrosion Resistant and Functional Materials, Jinan University, Guangzhou, Guangdong 523808, China

[5] Department of Materials Science and Engineering, College of Engineering, City University of Hong Kong, Tat Chee Avenue, Kowloon Tong, Kowloon, Hong Kong 999077, China

[6] Yuhua Institute of Advanced Materials, Baoji Xigong Titanium Alloy Products Co., Ltd, Baoji 721300, China

† These authors contribute equally to this work.

*Corresponding author. E-mail: yonyang@cityu.edu.hk, shijzhao@cityu.edu.hk, zhejia@seu.edu.cn.

SUPPLEMENTARY MATERIALS

Figure S1 to S18 Table S1 to S5

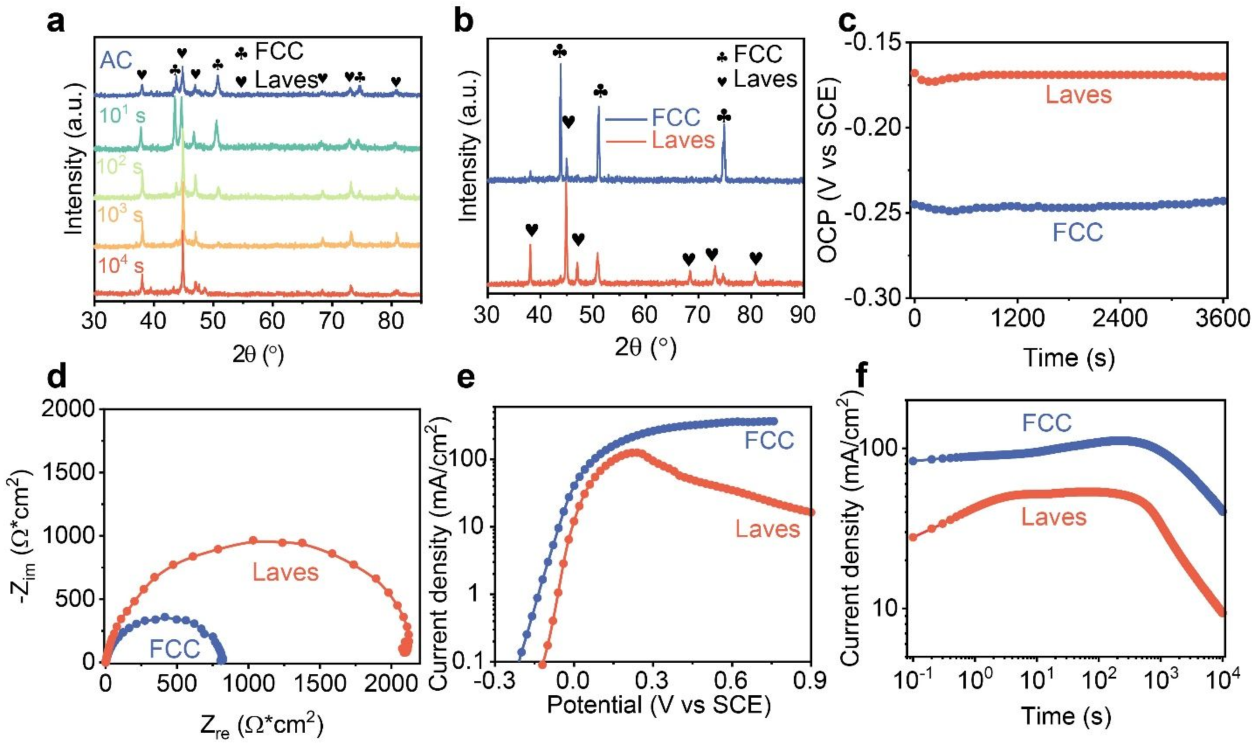

Figure S1. Thermodynamics of eutectic multi-principal element alloy (EMPEA) dealloying. (a) X-ray diffraction (XRD) patterns of EMPEA with different dealloying time 0 s, 10 s, 10^2 s, 10^3 s and 10^4 s. (b) The XRD pattern of the single-phase FCC and Laves. (c) Open circuit potential (OCP) of the FCC and Laves. (d) Nyquist plots of the FCC and Laves, performed at the corresponding OCP with the applied alternating current (AC) amplitude of 10 mV in the frequency range of 100 kHz to 10 mHz. (e) Potential dynamic polarization (PDP) of the FCC and Laves. (f) Potentiostatic polarization curves of FCC and Laves at 0.1 V vs SCE in 1 M HCl.

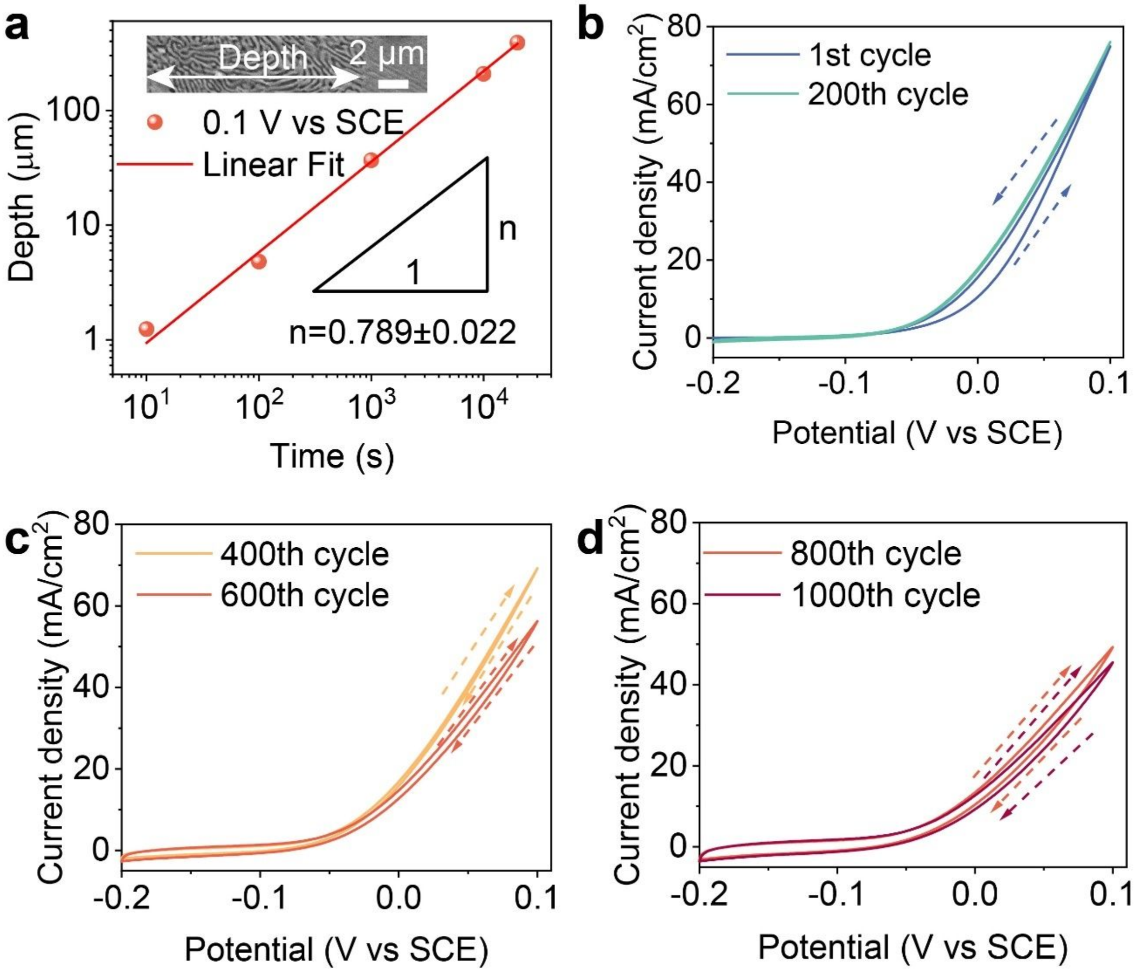

Figure S2. Kinetics of EMPEA dealloying. (a) Dealloying depth against time, inset is the SEM image of dealloying depth. (b-d) CV curves at different cycles.

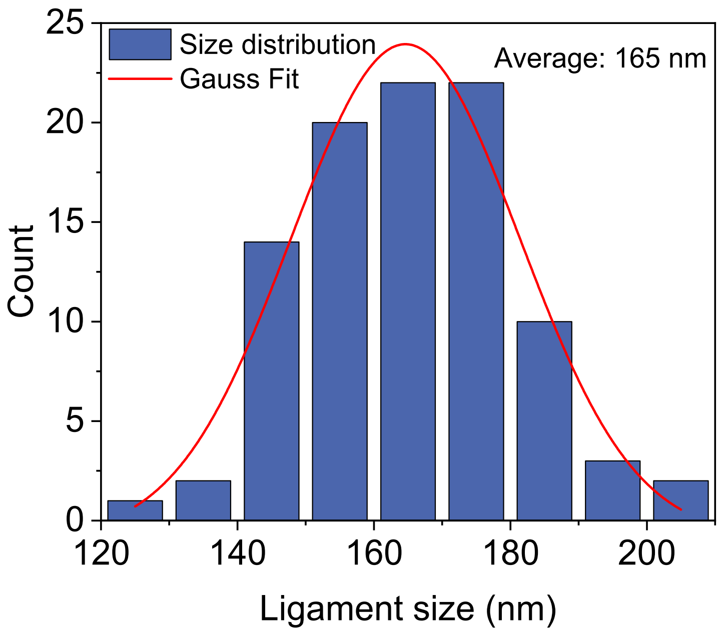

Figure S3. Size distribution of our nanostructured MPEA, with average size to be 165 nm.

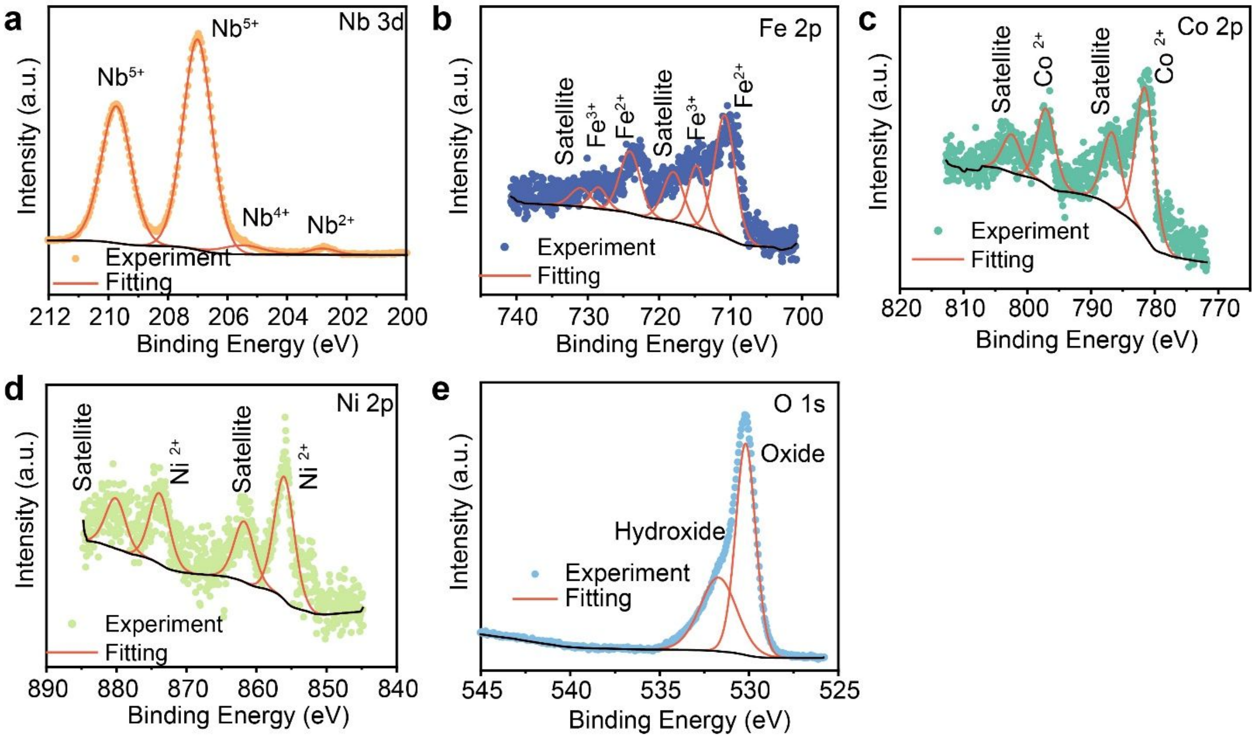

Figure S4. High-resolution X-ray photoelectron spectroscopy (XPS) spectra with peak deconvolution for the 10^4 s dealloyed EMPEA: (a) Nb 3d, (b) Fe 2p, (c) Co 2p, (d) Ni 2p, and (e) O 1s core-level regions.

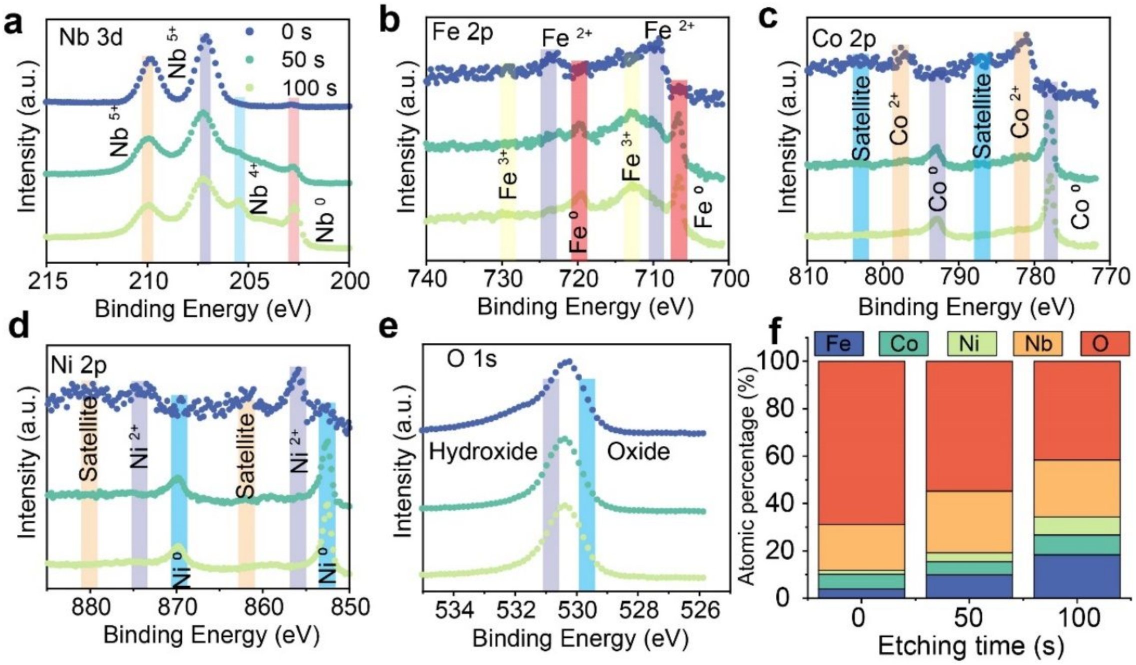

Figure S5. X-ray photoelectron spectroscopy of 10^4 s dealloyed EMPEA. (a-e) (XPS) depth profile analysis of the dealloyed sample, Narrow-scan XPS spectra for (a) Nb 3d, (b) Fe 2p, (c) Co 2p, (d) Ni 2p and (e) O 1s as a function of etching time. (f) Relative atomic concentration of Fe, Co, Ni, Nb and O with etching time, as obtained from the quantitative analysis of the XPS.

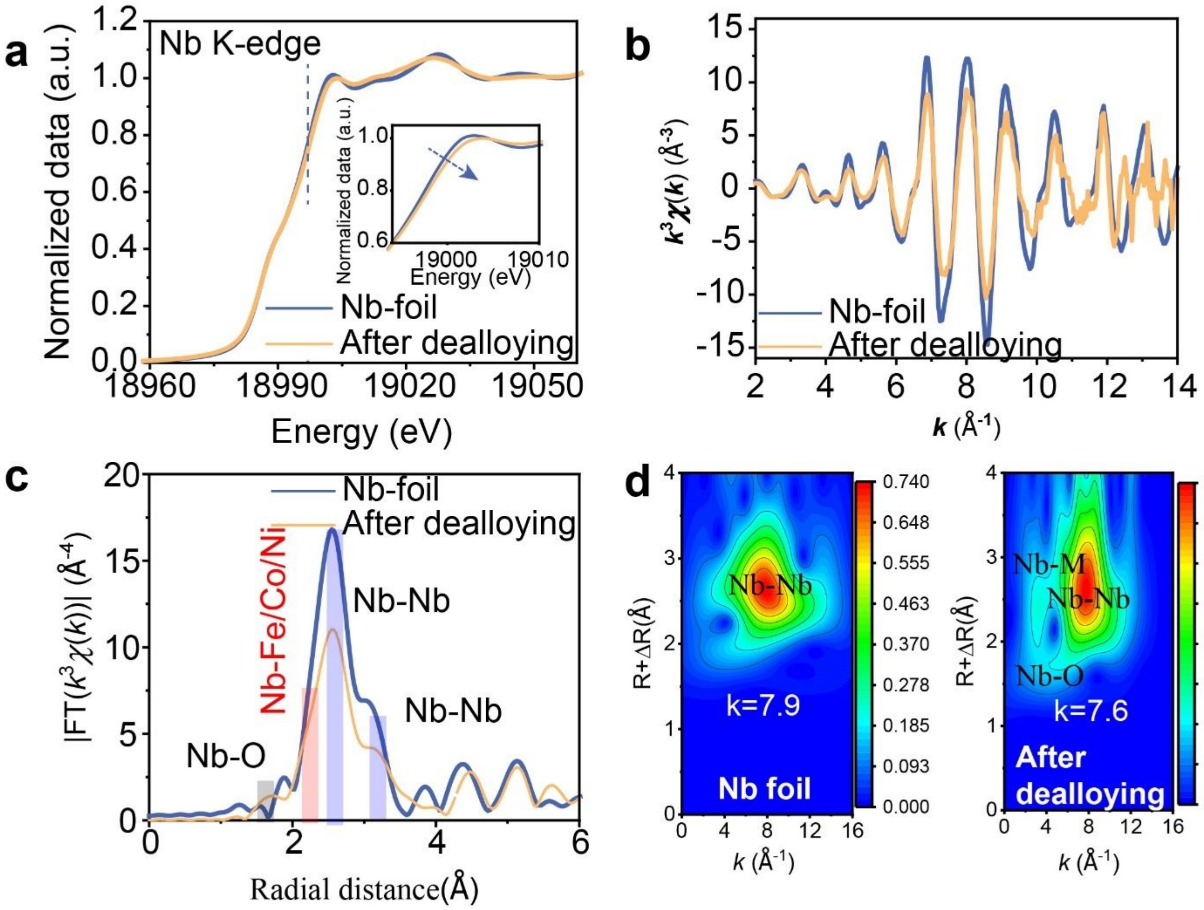

Figure S6. X-ray absorption spectroscopy (XAS) of 10^4 s dealloyed EMPEA. (a) X-ray absorption near edge structure (XANES) at Nb K-edge. (b) EXAFS χ(k) signals in k-space. (c) FT-EXAFS region for the local structure of Nb. (d) show the wavelet transform for the k3-weighted EXAFS Nb K-edge signal of Nb foil and dealloyed EMPEA, respectively.

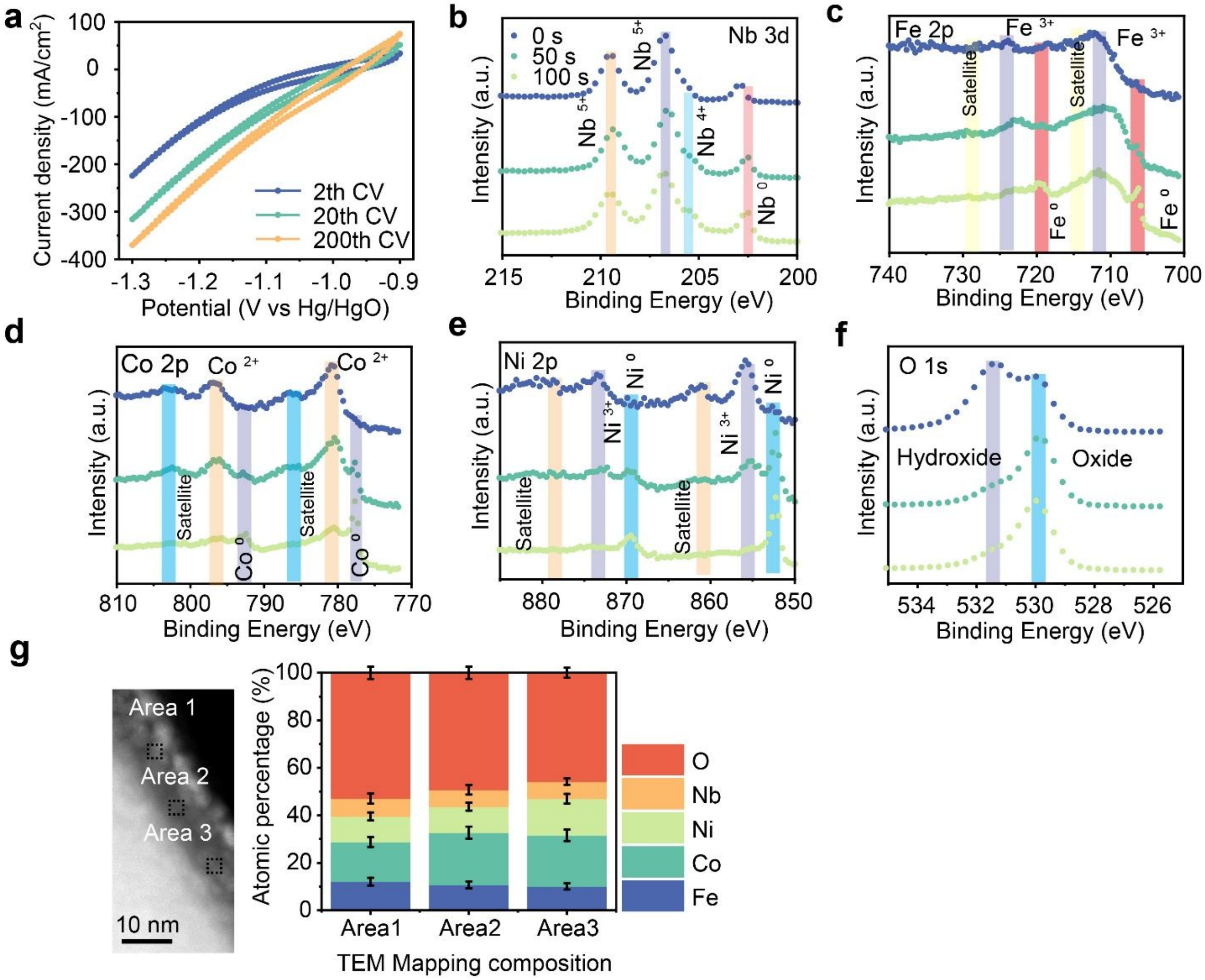

Figure S7. Characterization of CV activated 10^4 s dealloyed EMPEA. (a) CV curves [-0.9 - -1.3] V at different cycles. (b-f) XPS depth profile analysis of the dealloyed sample after CV activation, Narrow-scan XPS spectra for (b) Nb 3d, (c) Fe 2p, (d) Co 2p, (e) Ni 2p, and (f) O 1s as a function of etching time. (g) TEM composition of the oxide layer at three different areas.

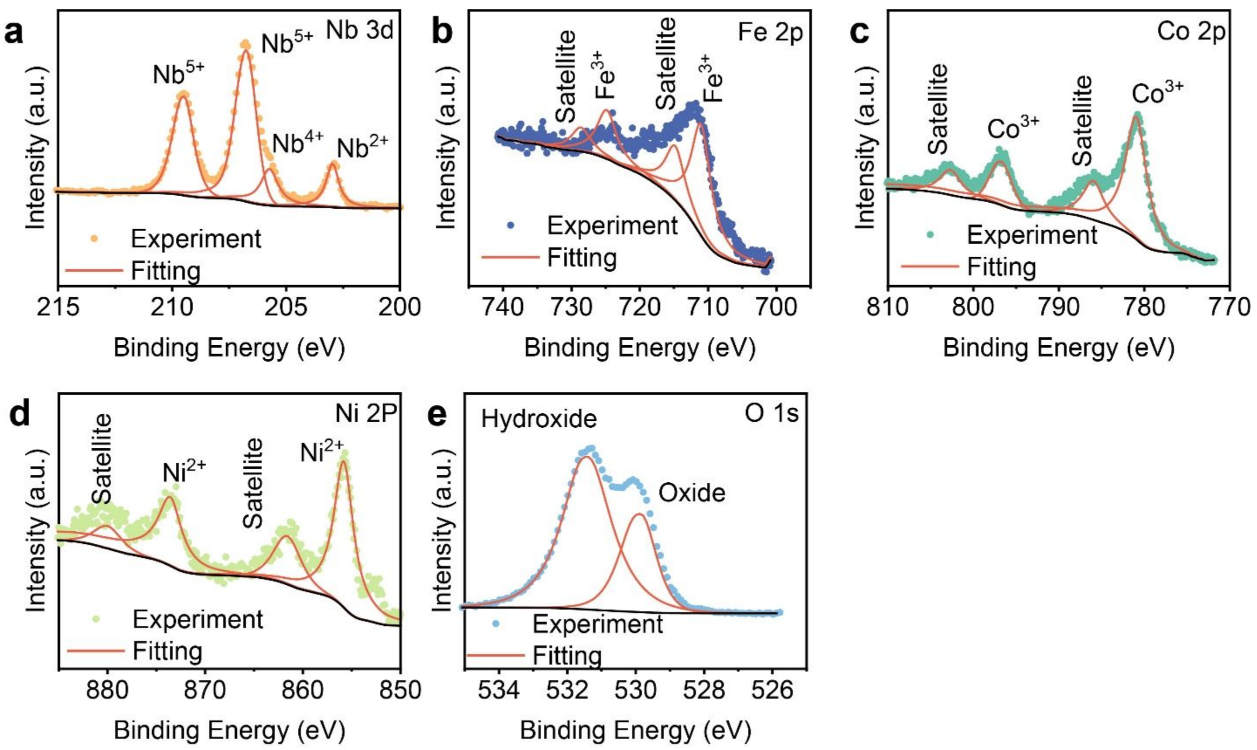

Figure S8. High-resolution XPS spectra with peak deconvolution of CV-activated 10^4 s dealloyed EMPEA surface: (a) Nb 3d, (b) Fe 2p, (c) Co 2p, (d) Ni 2p, and (e) O 1s core-level regions.

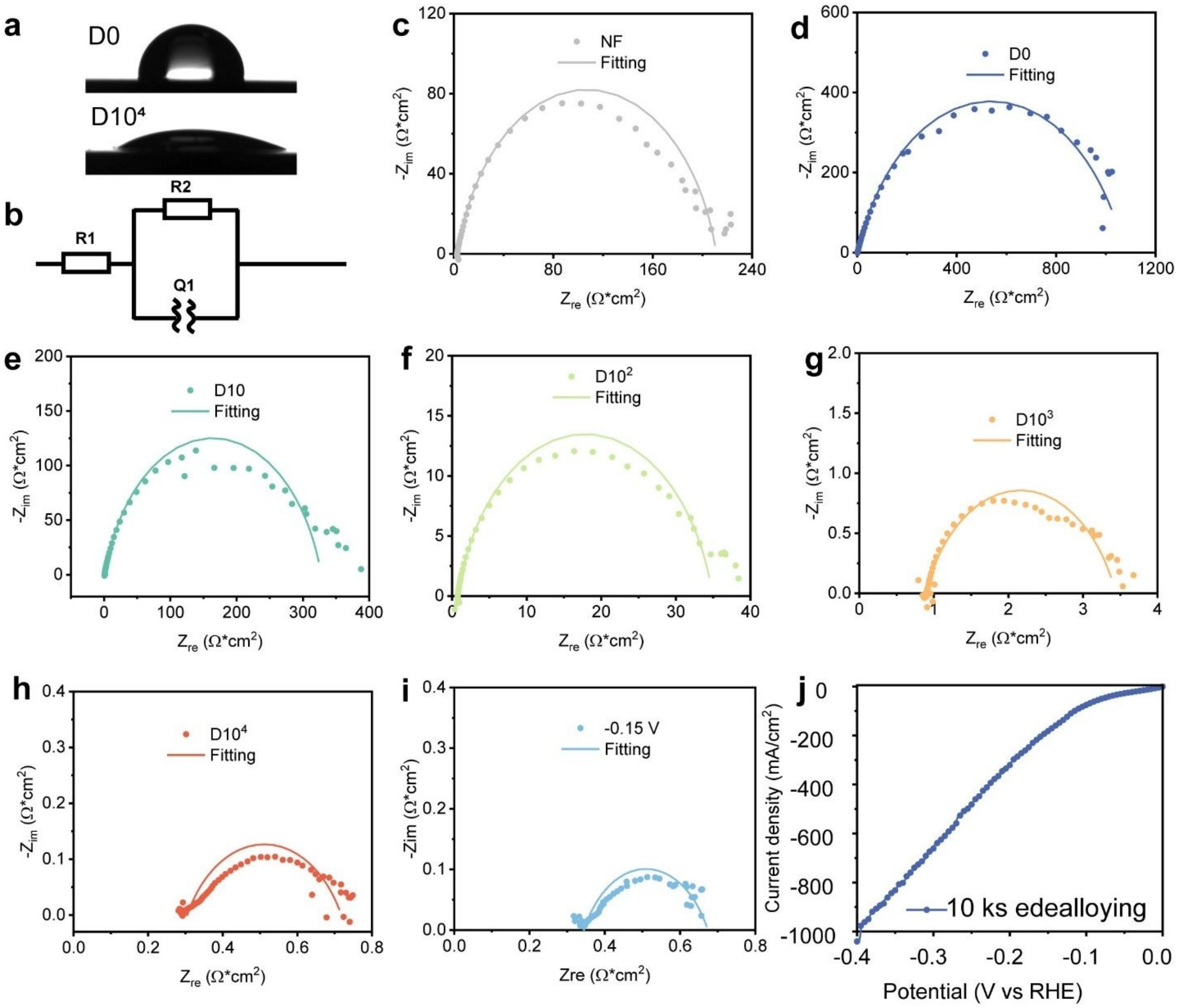

Figure S9. (a) Water contact angle of D0 and D10^4 samples (b) The equivalent circuit for EIS fitting. (c) Nyquist plots of Nickel Foam and (d-h) EMPEA at different dealloying time, performed at the -0.1 V vs RHE with the applied alternating current (AC) amplitude of 10 mV in the frequency range of 100 kHz to 10 mHz. (i) Nyquist plots of EMPEA at 10^4 s dealloying time, performed at the -0.15 V vs RHE with the applied alternating current (AC) amplitude of 10 mV in the frequency range of 100 kHz to 10 mHz in comparison with g, showing smaller semicircle diameter, thus smaller transfer resistance. (j) Linear sweep voltammetry (LSV) curves of 10^4 s dealloyed samples, with which we can see that we need ~ 396 mV to research 1000 mA/cm^2.

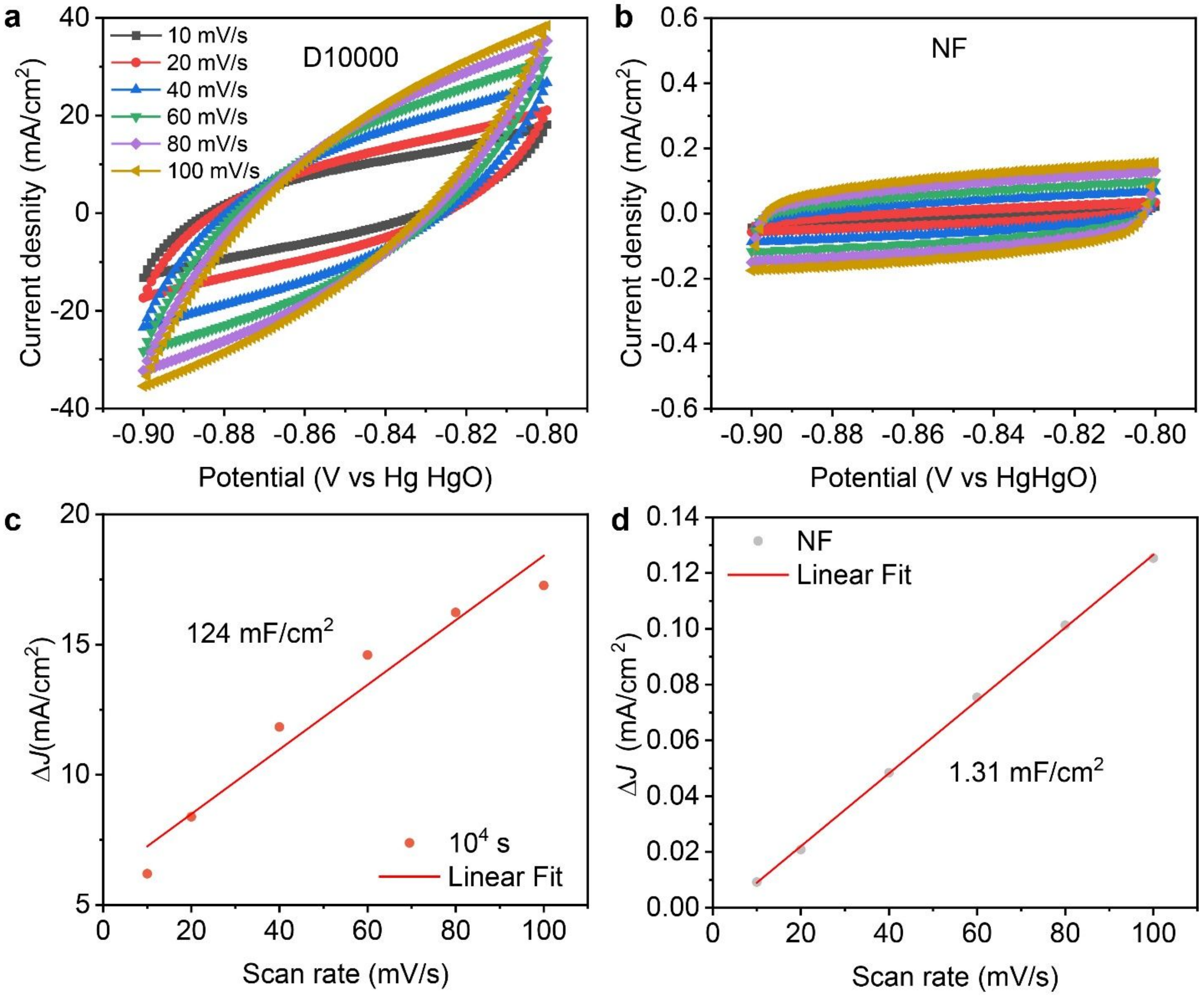

Figure S10. ECSA estimation of dealloyed nanostructured MPEA and NF. (a, b) Cyclic voltammetry of D10^4 nanostructured MPEA and NF at various scan rates (10 mV/s, 20 mV/s, 40 mV/s, 60 mV/s, 80 mV/s and 100 mV/s). (c, d) The plots of current densities against scan rates.

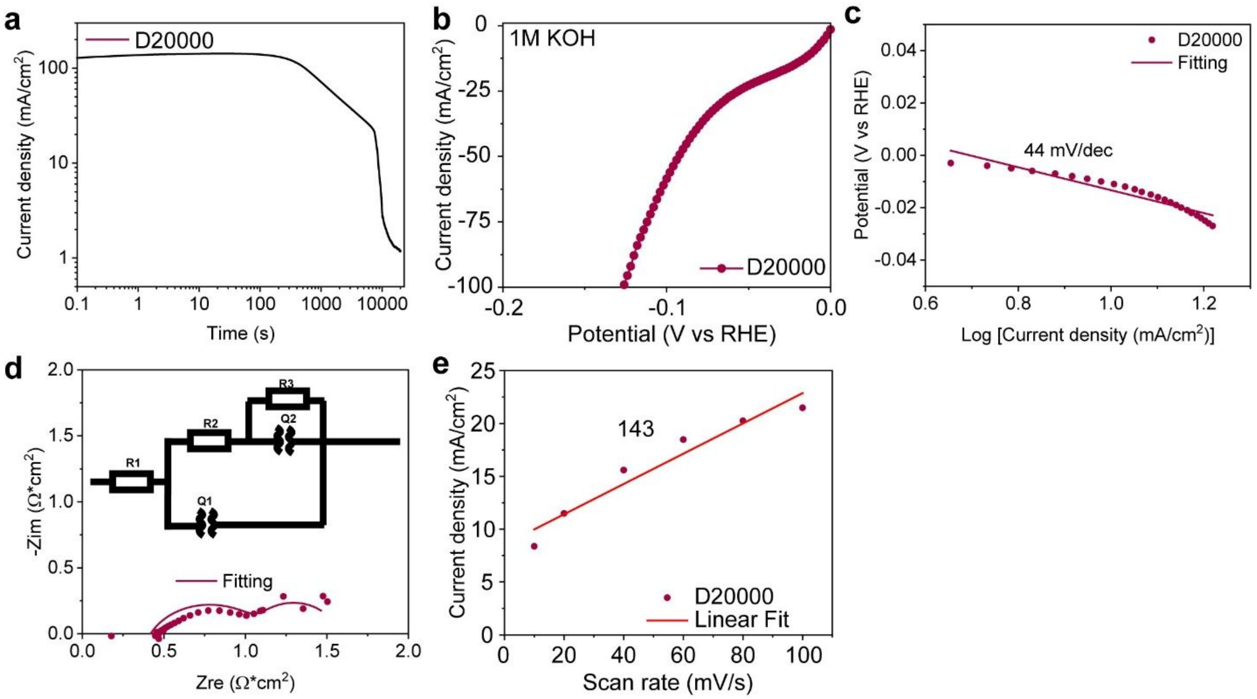

Figure S11. Alkaline HER performance of EMPEA at 20ks dealloying. (a) Potentiostatic polarization curves of EMPEA at 0.1 V vs SCE in 1 M HCl. (b) Linear sweep voltammetry (LSV) curves. (c) Tafel slopes. (d) Nyquist plots, performed at the -0.1 V vs RHE with the applied alternating current (AC) amplitude of 10 mV in the frequency range of 100 kHz to 10 mHz. (e) ECSA.

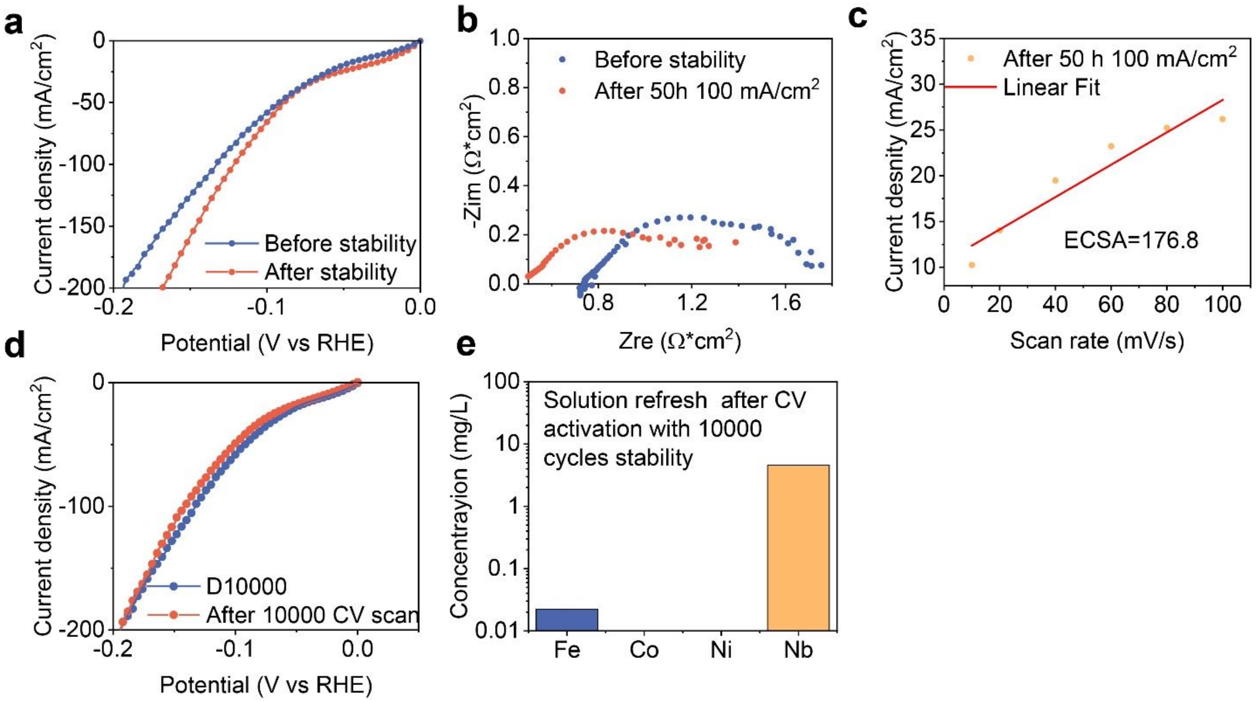

Figure S12. Alkaline HER performance of 10^4 s dealloyed EMPEA after stability tests. (a) Linear sweep voltammetry (LSV) curves before and after stability test. (b) Nyquist plots before and after stability tests, performed at the -0.1 V vs RHE with the applied alternating current (AC) amplitude of 10 mV in the frequency range of 100 kHz to 10 mHz. (c) ECSA after stability test. (d) Linear sweep voltammetry (LSV) curves before and after 10000 cycles CV. (e) ICP-OES after 10000 cycles CV stability.

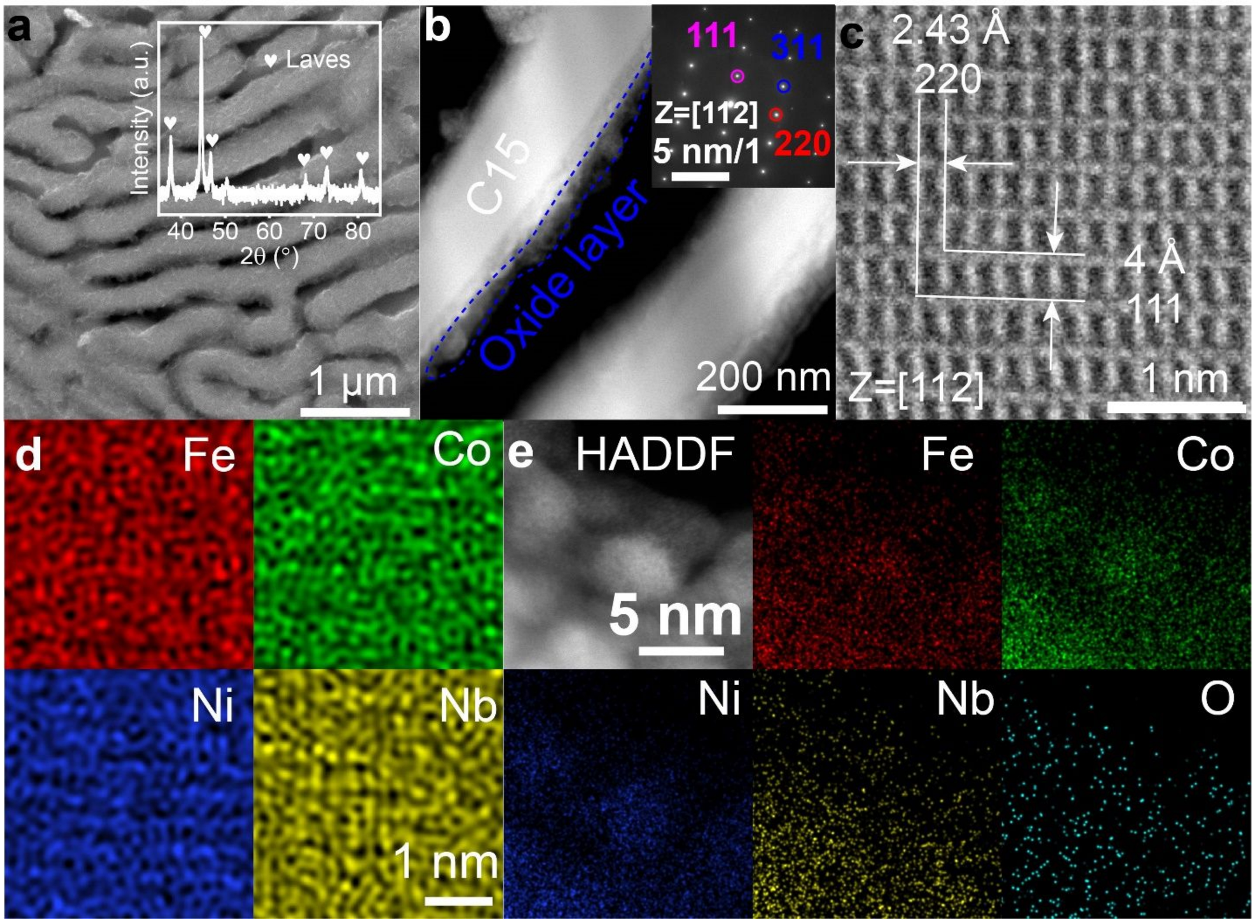

Figure S13. Structural characterization of 10^4 s dealloyed EMPEA after 50 hours 100 mA/cm^2 stability test. (a) SEM images, inset shows the GXRD pattern. (b) Low-magnification TEM image of the dealloyed eutectic alloy, inset shows the SADP. (c-d) High-magnification HAADF-STEM image accompanied by atomic-resolution elemental maps clearly showing the ordered crystal-lographic structure and site occupancy of the Laves structure. (e) HADDF images and Elements maps of Fe, Co, Ni, Nb and O.

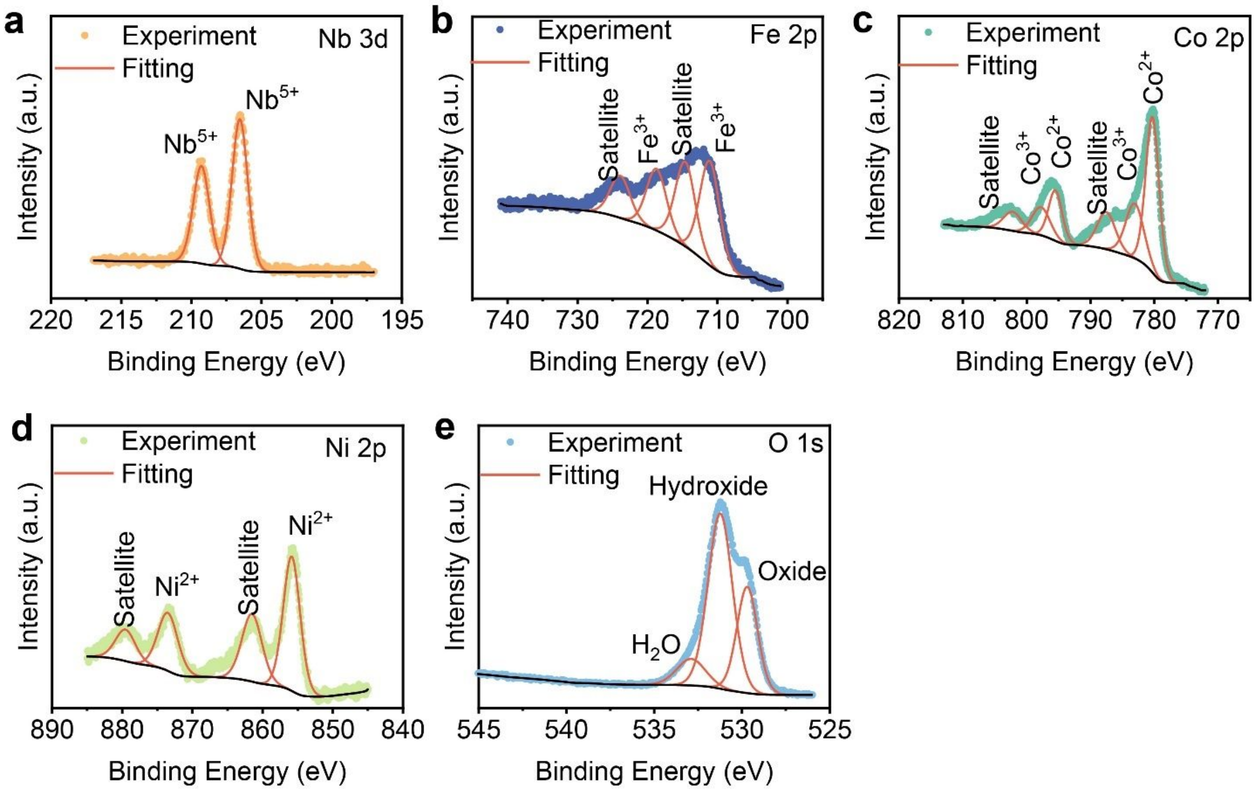

Figure S14. High-resolution X-ray photoelectron spectroscopy (XPS) analysis of the dealloyed EMPEA after 50 h stability testing. Deconvoluted core-level spectra of (a) Nb 3d, (b) Fe 2p, (c) Co 2p, (d) Ni 2p, and (e) O 1s regions.

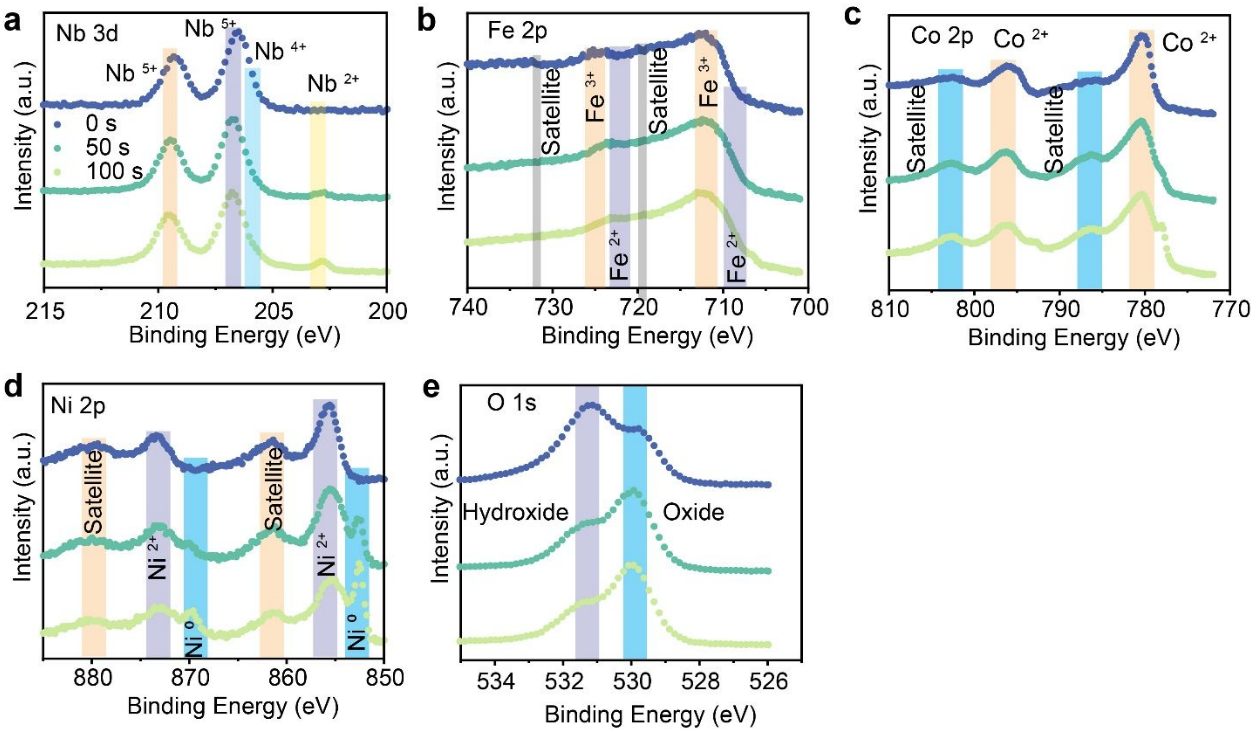

Figure S15. XPS depth profile analysis of the 10^4 s dealloyed EMPEA after 50 h stability, Narrow-scan XPS spectra for (a) Nb 3d, (b) Fe 2p, (c) Co 2p, (d) Ni 2p, and (e) O 1s as a function of etching time.

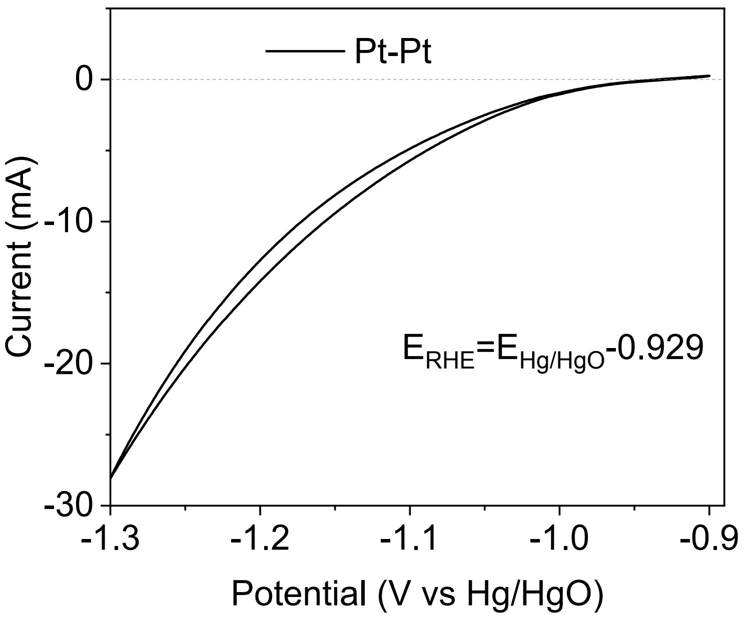

Figure S16. Calibration of the The Hg/HgO electrode with an H_2 atmosphere.

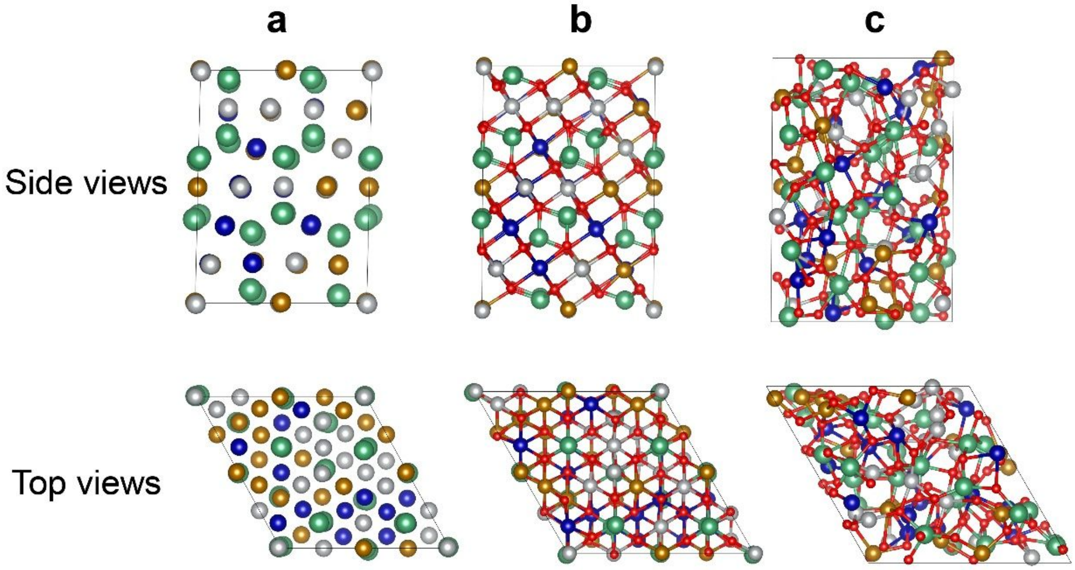

Figure S17. Atomic structure models of (a) C15 intermetallic phase, (b) C15-type crystalline oxide, and (c) amorphous oxide, depicted in top and side views.

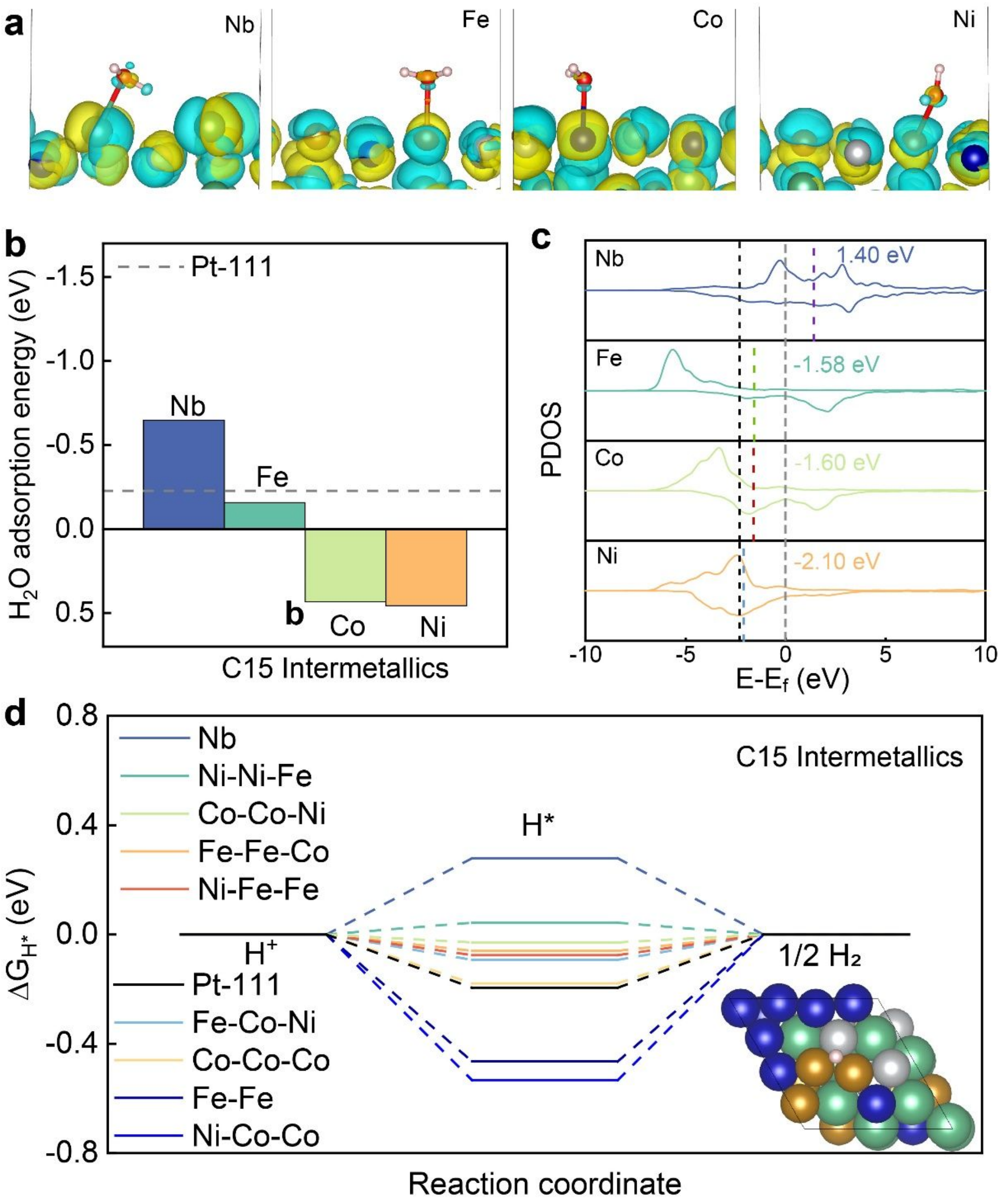

Figure S18. The atomic and electronic origin of excellent HER activity of C15 intermetallics. (a) The charge density difference before and after the adsorption of H_2O in C15 intermetallics. The blue and yellow isosurfaces correspond to charge densities of 0.01 e/Å^3 and -0.01 e/Å^3, respectively. (b) DFT calculated E_{ad} of H_2O molecules on the surfaces of the C15 intermetallics, in comparison with Pt-(111), revealing enhanced H_2O molecule adsorption on the catalyst surface in the Volmer step of HER. (c) PDOS on C15 intermetallics at crystal orientation (111). (d) Adsorption free energy versus the reaction coordinate of HER for C15 intermetallics.

Table S1. EXAFS data fitting results of Sample after dealloying with Nb foil as a reference.

Sample	Path	CN^a	R(Å)^b	σ^2 (Å^2)^c	ΔE_0(eV)^d	R factor
Nb K-edge (S_0^2=0.960)						
Nb foil	Nb-Nb	8*	2.855±0.003	0.0069	5.0	0.0037
	Nb-Nb	6*	3.290±0.005	0.0076		
After dealloying	Nb-M	1.2±0.3	2.702±0.026	0.0070	-3.5	0.0032
	Nb-Nb	6.9±0.4	2.871±0.011	0.0054	7.9	
	Nb-Nb	3.6±0.4	3.314±0.015			

$^a CN$, coordination number; $^b R$, the distance between absorber and backscatter atoms; $^c \sigma^2$, the Debye Waller factor value; $^d \Delta E_0$, inner potential correction to account for the difference in the inner potential between the sample and the reference compound; R factor indicates the goodness of the fit. $S0^2$ was fixed to 0.960, according to the experimental EXAFS fit of Nb foil by fixing CN as the known crystallographic value. * This value was fixed during EXAFS fitting, based on the known structure of Nb. Fitting conditions: k range：2.0 - 13.0; R range: 1.4-3.5; fitting space: R space; k-weight = 3. A reasonable range of EXAFS fitting parameters: $0.800 < S_0^2 < 1.000$; $CN > 0$; $\sigma^2 > 0$ Å^2; $|\Delta E_0| < 15$ eV; R factor < 0.02.

Table S2. Equivalent circuit elements values for EIS data (Figure S9) corresponding to NF and EHEA with different dealloying time and applied potential in 1M KOH solution.

	R1 ((Ω cm^2)	Error%	R2 (Ω cm^2)	Error%	Q1 (Ω^{-1} cm^{-2} s^{-n})	Error%	n	Error %
NF	2.69	3.11	209.00	3.90	1.10E-03	7.68	0.85	1.88
D0	0.72	4.59	1073.70	5.60	9.60E-04	5.23	0.78	1.23
D10	0.61	5.60	327.00	6.00	1.23E-03	8.43	0.83	1.84
$D10^2$	0.74	3.56	34.17	5.76	0.02	8.60	0.85	2.74
$D10^3$	0.89	0.84	2.55	2.47	0.19	4.67	0.75	2.39
$D10^4$ (0.1 V)	0.30	0.93	0.41	2.94	0.48	9.66	0.70	4.07
$D10^4$ (0.15 V)	0.34	0.93	0.33	3.28	0.46	12.60	0.70	5.01

Table S3. Comparison of the HER performance and stability for the recently reported electrocatalysts in alkaline media.

Material	Tafel (mV/dec)	η10 (mV)	Time (h)	Current density (mA/cm^2)	Reference
MnNiCuCoVFe MoPdPtAuRuIr	29.5	21	100	400	[1]
PtPdNiP	40	19.8	60	20	[2]
FeCoNiPB	205	163	-	-	[3]
FeCoNiAlTi	40.1	88.2	40	100	[4]
FeCoNiCu	31.7	42.2	120	500	[5]
NiCuPtPdAu	47	38	-	-	[6]
NiMnFeMo	32	28	180	100	[7]
PdPtCuNiP	35	32	95	20	[8]
VSnFeCoNiC	38	172	-	-	[9]
FeCoNiCuAl	44	68	60	50	[10]
FeCoNiCrAlW	112	101	60	-	[11]
FeCoNiBPt	30.9	18	200	100	[12]
CuFeZnAgAu	96	282	-	-	[13]
FeCoNiMnP	33.5	43	30	50	[14]
FeCoNiCrCu	62	84	14	10	[15]
FeCoNiNb	77	22	553	1000	This work
FeCoNiNb	116	362	-	-	This work
Nickel Foam	100	273	-	-	This work

Table S4. ΔG_{H*} on the surface of C15 crystalline oxides.

Adsorption sites	ΔG_{H*} (eV)	Adsorption sites	ΔG_{H*} (eV)
Nb	-0.138	**Fe**	**0.350**
Nb	0.556	Fe	0.432
Nb	0.976	Fe	-2.220
Nb	-0.865	Fe	1.063
Nb	-0.636	Co	0.652
Nb	0.165	**Co**	**0.371**
Nb	-0.093	Co	1.501
**Nb**	**-0.043**	Co	1.457

Table S5. ΔG_{H*} on the surface of amorphous oxides.

Adsorption sites	ΔG_{H*} (eV)	Adsorption sites	ΔG_{H*} (eV)
**Nb**	**0.203**	Co	1.016
Nb	0.573	Co	0.896
Nb	0.221	Co	1.582
Nb	1.295	Co	1.068
Nb	0.408	**Co**	**0.532**
**Fe**	**-0.599**	**Ni**	**0.316**
Fe	2.018	Ni	1.412
Fe	1.381	Ni	1.250
Co	0.554	Ni	1.702
Co	0.907	Ni	0.729
Co	1.250	Ni	1.939
Ni	1.463	FeCo	**0.074**
**NbNi**	**0.317**	NiFe	**-0.416**
**NbCo**	**0.246**	NbCo	1.254

References

(1) Yu, T.; Zhang, Y.; Hu, Y.; Hu, K.; Lin, X.; Xie, G.; Liu, X.; Reddy, K. M.; Ito, Y.; Qiu, H. J. Twelve-Component Free-Standing Nanoporous High-Entropy Alloys for Multifunctional Electrocatalysis. *ACS Mater. Lett.* **2022**, *4* (1), 181–189. https://doi.org/10.1021/acsmaterialslett.1c00762.

(2) Jia, Z.; Yang, Y.; Wang, Q.; Kong, C.; Yao, Y.; Wang, Q.; Sun, L.; Shen, B.; Kruzic, J. J. An Ultrafast and Stable High-Entropy Metallic Glass Electrode for Alkaline Hydrogen Evolution Reaction. *ACS Mater. Lett.* **2022**, *4* (8), 1389–1396. https://doi.org/10.1021/acsmaterialslett.2c00371.

(3) Wei, R.; Zhang, K.; Zhao, P.; An, Y.; Tang, C.; Chen, C.; Li, X.; Ma, X.; Ma, Y.; Hao, X. Defect-Rich FeCoNiPB/(FeCoNi)3O4-x High-Entropy Composite Nanoparticles for Oxygen Evolution Reaction: Impact of Surface Activation. *Appl. Surf. Sci.* **2021**, *549* (February), 149327. https://doi.org/10.1016/j.apsusc.2021.149327.

(4) Jia, Z.; Yang, T.; Sun, L.; Zhao, Y.; Li, W.; Luan, J.; Lyu, F.; Zhang, L.; Kruzic, J. J.; Kai, J.; Huang, J. C.; Lu, J.; Liu, C. T. A Novel Multinary Intermetallic as an Active Electrocatalyst for Hydrogen Evolution. *Adv. Mater.* **2020**, *32* (21), 1–9. https://doi.org/10.1002/adma.202000385.

(5) Li, R.; Liu, X.; Liu, W.; Li, Z.; Chan, K. C.; Lu, Z. Design of Hierarchical Porosity Via Manipulating Chemical and Microstructural Complexities in High-Entropy Alloys for Efficient Water Electrolysis. *Adv. Sci.* **2022**, *9* (12), 1–10. https://doi.org/10.1002/advs.202105808.

(6) Qiu, H. J.; Fang, G.; Wen, Y.; Liu, P.; Xie, G.; Liu, X.; Sun, S. Nanoporous High-Entropy Alloys for Highly Stable and Efficient Catalysts. *J. Mater. Chem. A* **2019**, *7* (11), 6499–6506. https://doi.org/10.1039/c9ta00505f.

(7) Liu, H.; Xi, C.; Xin, J.; Zhang, G.; Zhang, S.; Zhang, Z.; Huang, Q.; Li, J.; Liu, H.; Kang, J. Free-Standing Nanoporous NiMnFeMo Alloy: An Efficient Non-Precious Metal Electrocatalyst for Water Splitting. *Chem. Eng. J.* **2021**, *404* (June 2020), 126530. https://doi.org/10.1016/j.cej.2020.126530.

(8) Jia, Z.; Nomoto, K.; Wang, Q.; Kong, C.; Sun, L.; Zhang, L. C.; Liang, S. X.; Lu, J.; Kruzic, J. J. A Self-Supported High-Entropy Metallic Glass with a Nanosponge Architecture for Efficient Hydrogen Evolution under Alkaline and Acidic Conditions. *Adv. Funct. Mater.* **2021**, *31* (38), 1–12. https://doi.org/10.1002/adfm.202101586.

(9) Yang, J.; Fan, Y.; Liu, Y.; Zhang, C.; Zou, H.; Xiong, L.; Li, X. Self-Supporting Porous High-Entropy MAX Electrode for Highly Active Electrocatalyst H2 Evolution in Alkali Solution. *J. Porous Mater.* **2022**, *29* (3), 693–704. https://doi.org/10.1007/s10934-022-01205-5.

(10) Yang, Y.; Jia, Z.; Zhang, X.; Liu, Y.; Wang, Q.; Li, Y.; Shao, L.; Di, S.; Kuang, J.; Sun, L.; Zhang, L. C.; Kruzic, J. J.; Lu, Y.; Lu, J.; Shen, B. Chemical Short-Range Order in Multi-Principal Element Alloy with Ordering Effects on Water Electrolysis Performance. *Mater. Today* **2024**, *72* (xx), 97–108. https://doi.org/10.1016/j.mattod.2023.12.006.

(11) Han, X.; Chen, Q.; Chen, Q.; Wu, Q.; Xu, Z.; Zheng, T.; Li, W.; Cui, D.; Duan, Z.; Zhang,

J.; Li, J.; Li, H.; Wang, Z.; Wang, J.; Xia, Z. Eutectic Dual-Phase Microstructure Modulated Porous High-Entropy Alloys as High-Performance Bifunctional Electrocatalysts for Water Splitting. *J. Mater. Chem. A* **2022**, *10* (20), 11110–11120. https://doi.org/10.1039/d2ta01701f.

(12) Zhang, X.; Yang, Y.; Liu, Y.; Jia, Z.; Wang, Q.; Sun, L.; Zhang, L.; Kruzic, J. J.; Lu, J.; Shen, B. Defect Engineering of a High-Entropy Metallic Glass Surface for High-Performance Overall Water Splitting at Ampere-Level Current Densities. *Adv. Mater.* **2023**, *2303439*, 1–11. https://doi.org/10.1002/adma.202303439.

(13) Praveen Kumar, S.; Sharafudeen, P. C.; Elumalai, P. High Entropy Metal Oxide@graphene Oxide Composite as Electrocatalyst for Green Hydrogen Generation Using Anion Exchange Membrane Seawater Electrolyzer. *Int. J. Hydrogen Energy* **2023**, *48* (97), 38156–38171. https://doi.org/10.1016/j.ijhydene.2023.06.121.

(14) Zhang, H.-M.; Zuo, L.; Gao, Y.; Guo, J.; Zhu, C.; Xu, J.; Sun, J. Amorphous High-Entropy Phosphoxides for Efficient Overall Alkaline Water/Seawater Splitting. *J. Mater. Sci. Technol.* **2024**, *173*, 1–10. https://doi.org/10.1016/j.jmst.2023.08.003.

(15) Wang, Y.; Yang, H.; Zhang, Z.; Meng, X.; Cheng, T.; Qin, G.; Li, S. Far-from-Equilibrium Electrosynthesis Ramifies High-Entropy Alloy for Alkaline Hydrogen Evolution. *J. Mater. Sci. Technol.* **2023**, *166*, 234–240. https://doi.org/10.1016/j.jmst.2023.05.040.
